# Polaritonic Chemistry from First Principles via Embedding Radiation Reaction

**DOI:** 10.1021/acs.jpclett.2c01169

**Published:** 2022-07-22

**Authors:** Christian Schäfer

**Affiliations:** Department of Microtechnology and Nanoscience, MC2, Chalmers University of Technology, 412 96 Göteborg, Sweden

## Abstract

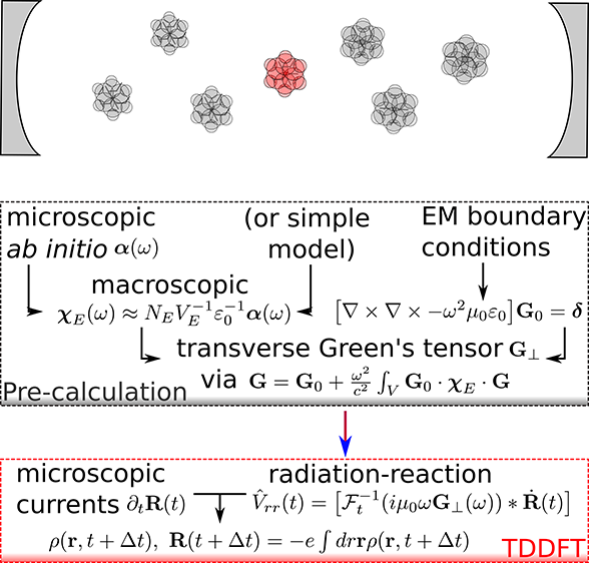

The coherent interaction of a large collection of molecules with a common photonic mode results in strong light-matter coupling, a feature that has proven highly beneficial for chemistry and has introduced the research topics polaritonic and QED chemistry. Here, we demonstrate an embedding approach to capture the collective nature while retaining the full *ab initio* representation of single molecules—an approach ideal for polaritonic chemistry. The accuracy of the embedding radiation-reaction ansatz is demonstrated for time-dependent density-functional theory. Then, by virtue of a simple proton-tunneling model, we illustrate that the influence of collective strong coupling on chemical reactions features a nontrivial dependence on the number of emitters and can alternate between strong catalyzing and an inhibiting effect. Bridging classical electrodynamics, quantum optical descriptions, and the *ab initio* description of realistic molecules, this work can serve as a guiding light for future developments and investigations in the quickly growing fields of QED chemistry and QED material design.

The self-consistent interaction between light and matter developed over recent years into a viable and important tool to nonintrusively shape chemistry and materials on demand. Specific resonator geometries (cavities) support a limited set of eigenmodes which couple strongly to matter, resulting in new quasiparticles, so-called polaritons. Typical realizations of those resonators range between nanometer-sized plasmonic structures, that can couple to individual molecules, and Fabry-Pérot cavities, in which many molecules couple collectively.^1−6^ Such a hybridization between cavity mode(s) and matter exists also in the absence of driving, in contrast to Floquet physics, which is often limited by induced heating and decoherence^7,8^—the mere existence of the confined modes can alter materials. This includes the control of photochemical reactions by coupling to electronic excitations,^9−18^ ground-state chemical reactions via vibrational strong coupling,^19−24^ and energy and charge transfer over macroscopic dimensions.^25−31^ Furthermore, notable progress in the construction of cavities^10,32−36^ has led to a steadily rising number of applications outside chemistry, ranging from polariton-mediated lasing,^37−39^ over material design,^40−43^ to quantum information theory.^44−46^

However, most of the so far existing chemically relevant applications couple many molecules collectively to a common cavity mode. On one hand, a predictive theoretical study of chemical processes requires a thorough description of the electronic and vibrational structure from first principles. On the other hand, a direct evaluation of large ensembles is prohibited by the quickly increasing computational cost. The vast majority of theoretical studies are therefore based on simplified models that possess a limited applicability to complex systems and chemical reactions. Descriptions from first-principles have been restricted to small numbers of molecules.^47−53^ Notable representatives are quantum-electrodynamical density-functional theory (QEDFT)^54−57^ and cavity coupled-cluster theory.^58,59^

By embedding the majority of the molecular ensemble into a local potential, we will discover a path to open first-principles techniques toward collective strong-coupling with arbitrary numbers of ensemble molecules and species. This approach represents an extension of the radiation-reaction ansatz derived in reference (47) and inherits its computational and conceptual simplicity, allowing an almost effortless implementation into existing time-dependent density-functional theory (TDDFT) libraries.

Electronic and nuclear dynamics influenced by light are governed by the nonrelativistic Hamiltonian: 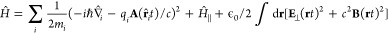
with fixed Coulomb gauge ∇·**A** = 0. The electromagnetic fields follow Maxwell’s equation; self-consistency emerges when light and matter are solved simultaneously. As demonstrated in reference (47), the classical interaction with light can be efficiently accounted for via the transverse component of the dyadic Green tensor **G**_⊥_ with **E**_r,⊥_(**r**,ω) = *i*μ_0_ω ∫_*V*_ d*r*′**G**_⊥_(**r**,**r**′,ω)·(−*e***j**(**r**′,ω)). The Green tensor is the formal solution of Helmholtz’s equation^60^
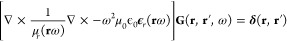
and characterizes the electromagnetic environment. The microscopic paramagnetic current **j** serves as driving inhomogeneity, i.e., oscillating charges emit light. In addition, we have the freedom to assume that parts of the system behave as a local and a potentially isotropic linear medium **ε**_r_ and μ_r_ which shape via the Helmholtz equation the electromagnetic environment, represented by **G**. In this way, **G** can subsume large fractions of a system while we free computational resources for the microscopic system described by **j**. The local radiation-reaction potential *V̂*_rr_(t) = −**R̂**·**E**_r,⊥_(*t*), with **E**_r,⊥_(*t*) = *F*_t_^–1^(*i*μ_0_ω**G**_⊥_(ω))∗**Ṙ**(*t*) and recalling that **Ṙ**(*t*) = ∫ d*r*(−*e***j**(**r***t*)), is obtained from the derivative of the dipole moment provided by the real-time TDDFT propagation. It acts on the microscopic currents via the Schrödinger equation and manifests the self-consistent interaction with transverse fields within the long-wavelength approximation. This allows us to extend, for example, TDDFT with the self-consistent classical interaction between an electromagnetic environment and matter by simply adding *v*_rr_(**r***t*) = *e***r**·E_*r*,⊥_(*t*) to the usual Kohn–Sham equations. We will focus in the following on polaritonic chemistry, i.e., electromagnetic environments that embody Fabry–Pérot-like eigenmodes and a large number of emitters. Clearly, **G**, and with it the presented embedding radiation-reaction ansatz, is generic and able to account for any kind of complex environment.^47^

In the collective strong-coupling regime between many molecules and a cavity, single photon processes dominate, and the individual effect of the cavity field acting on a single molecule is comparably weak. The electronic and vibrational excitation structure remains then largely unaffected; the combined light-matter system hybridizes based on those bare excitations (see SI for a detailed discussion). As long as this condition is satisfied, i.e., as long as light and matter do not couple ultra-strongly, it is safe to separate the matter ensemble into two parts. The first describes a single “impurity” molecule contributing the paramagnetic current **j**, which will be obtained as a solution to Schrödinger’s equation. The second accounts for all the remaining molecules as polarizable material **ε**_r_(**r**,ω) = **1** + **χ**_E_(**r**,ω). Surely this separation is also convenient to account for two different species, e.g., the reactive molecule and its solvent. In this sense, the problem of chemical reactions in collective light-matter coupling can be seen as an impurity problem; a single molecule undergoing a reaction is influenced by a large electromagnetic environment.

Knowing the bare Green tensor **G**_0_ (empty cavity), we can obtain the “ensemble-dressed” dyadic Green tensor with the help of the Dyson equation: 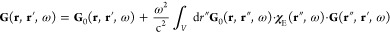
We assumed here (and earlier) that the generic susceptibility **P**(**r**,ω) = ε_0_ ∫ d*r*^3^**χ**(**r**,**r**′,ω)·**E**(**r**′,ω) is local **χ**(**r**,**r**′, ω) ≈ **χ**(**r**,ω)δ(**r** – **r**′). In many situations, the orientation of emitters within a gas or fluid will be random, and its average polarizability is consequentially isotropic.

For a dilute ensemble satisfying the long wavelength approximation, the widely believed regime of polaritonic chemistry, the response can be approximated as **χ**_E_(**r**^″^,ω) ≈ **χ**_E_(**r**_0_,ω)θ(**r** ∈ *V*_E_) ≈ **χ**_*E*_(ω)*V*_E_δ(**r** – **r**_0_). Then, 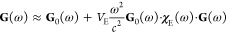
is evaluated at the position of the explicit molecule and *V*_E_ is the volume occupied by the ensemble of emitters. This simplifies further for the widely used one-dimensional Fabry–Pérot cavities
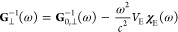
with **G**_⊥_(ω) ≈ **ϵ**_c_**ϵ**_c_^T^/[G_0_^–1^(ω) – ω^2^/*c*^2^*V*_E_**ϵ**_c_^T^·**χ**_E_(ω)·**ϵ**_c_]. Cavity losses are accounted for by adding a weak cavity-decay rate η. The remaining task is now to identify **χ**_E_(ω). The (dipolar) polarization can be conveniently obtained from the density–density response function^61^ as **R**(ω) = ∫ d*r*(−*e*)**r**δρ(**r**ω) = **α**(ω)·δ**E**_local_(ω) = ∫ d*r ∫ d*r′(−*e*^2^)**r**χ_ρρ_(**r**,**r**′,ω)**r**′·δ**E**_local_(ω), a standard task for any TDDFT code. The induced dipole is related to the polarization density **P**(ω) used in Maxwell’s equation:
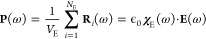
such that **χ**_E_(ω) = *N*_E_*V*_E_^–1^ϵ_0_^–1^**α**(ω), if **E** ≈ δ**E**_local_. This approximation is reasonable if we focus on weak external fields. Alternatively, the Clausius–Mossotti relation^62^ provides a simple and reliable solution for isotropic systems 
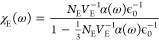
It might not always be possible or desirable to obtain the embedding kernel from first-principles, and instead simplified approaches such as the Drude-Lorentz model present easily accessible alternatives.

Equipped with the necessary framework, we are now able to describe the dynamic of single molecules in collective strong coupling relevant for polaritonic chemistry. We start by demonstrating the emergence of collective states for realistic systems from first-principles and validating the embedding radiation-reaction ansatz. We then shift our focus to chemistry and illustrate for a simple proton-tunneling model how chemical reactions are modified via collective vibrational strong-coupling.

The embedding radiation-reaction ansatz has been implemented into GPAW^63^—a simple task as the local potential is easily combined with the TDDFT framework. This allows us to validate the previous conceptions for a realistic system, here chosen to be a chain of sodium dimers in H-aggregate configuration oriented along the *z* direction. We follow the computational flow-chart illustrated in the table of contents (TOC) graphic with the simplification 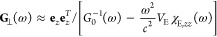
First, we obtain the bare response of a single sodium dimer with a width given by the radiation-reaction potential^47^ with the cross-sectional area *A* = 10^2^ Å^2^. Next, we obtain the polarizability α_*zz*_(ω) which defines **G**_⊥_(ω) and with it the corresponding embedding radiation-reaction potential. Finally, a linear-response calculation of a single sodium dimer that is affected by the ensemble-dressed cavity is performed.

Figure 1 sets the embedding approach (red) in relation with the direct response of N sodium dimers (black) coupled to the cavity mode. The spectra obtained from the embedding approach describe thus the response of a *single* molecule (e.g., the red molecule in the TOC graphic) and the spectral strength characterizes how strong this single molecule will contribute to the *collective* bright and dark polaritonic states. We expect therefore that the embedded response recovers the correct excitation poles of the collective states, but its spectral strength is that of the contribution of a single molecule to the collective state—precisely what Figure 1 demonstrates. The most notable difference is of course that the embedding approach describes the response of a single dimer, i.e., most of the spectral density is located in the dark state, which is not visible in the direct N-dimers + cavity calculations. The more molecules that couple collectively, the smaller (∝1/*N*, e.g., (2Na_2_)/(4Na_2_) ≈ 2) the contribution of a single molecule. Excitation energies and the asymmetry of the spectral weight are in excellent agreement. As expected, the embedding approach recovers the ‘with 1/*N* decreasing’ contribution of the single molecule to the collective state (see SI for an extended discussion). While the embedding approach sets its focus on the dynamic of single molecules, it allows recovery of the spectra of the full collective state (see SI for a detailed discussion). The direct calculations exhibit a weak blue-shift that can be removed by increasing the distance between dimers from 10 to 20 Å (magenta). Its origin lies in the Coulomb-mediated dipole–dipole interactions between the chain elements. Coulomb mediated couplings could be included in the longitudinal component of the embedding radiation-reaction ansatz or simply via commonly available techniques such as PCM or frozen-density embedding.^64−66^ This bright/dark state behavior represents the hallmark of collective strong coupling and is at the heart of polaritonic chemistry.^1,67^ However, if our single impurity molecule is detuned from the ensemble and cavity, the impurity will heavily out-weight all other molecules at the avoided crossing between impurity molecule and the polaritonic states (see SI and ref (68)).

**Figure 1:**
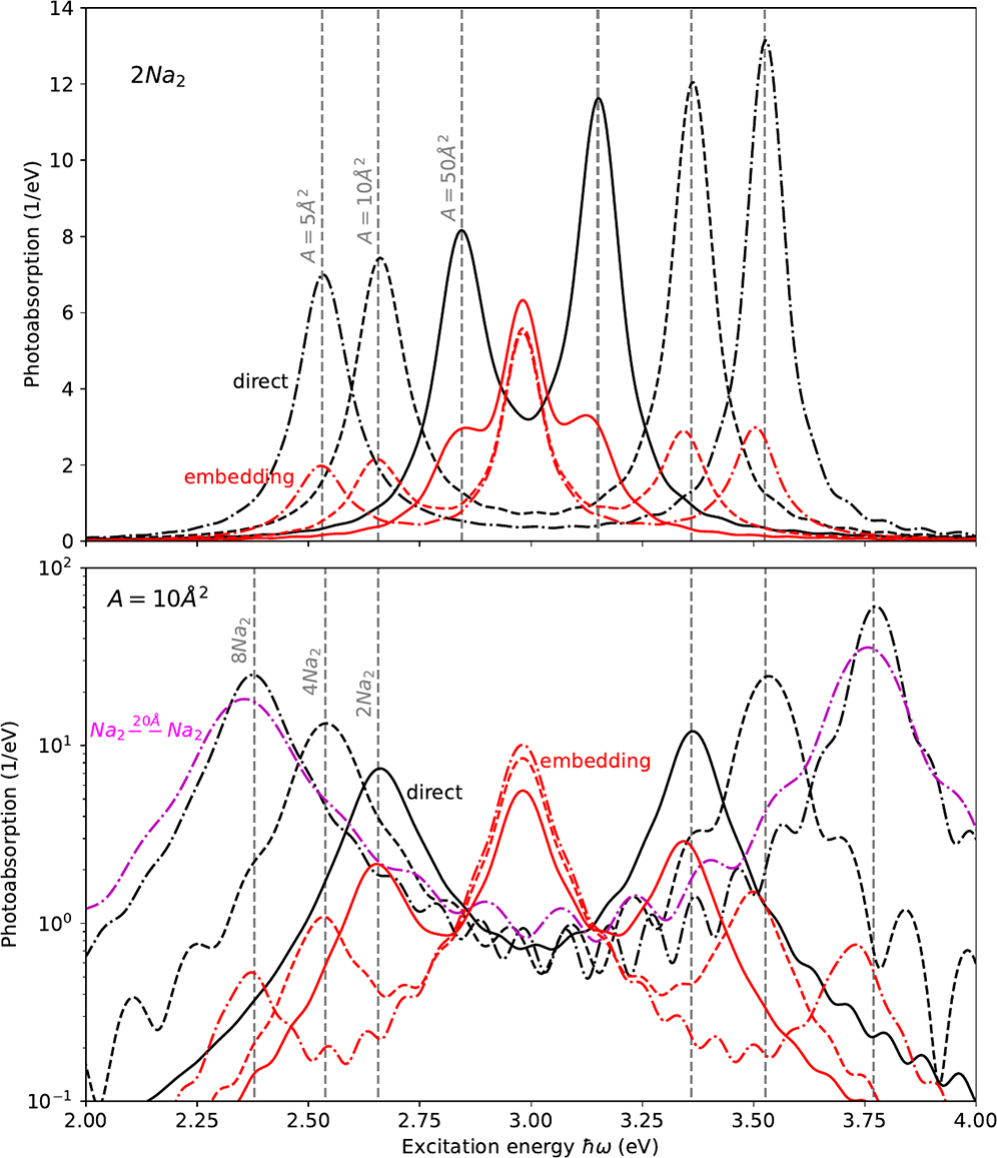
Photoabsorption of Na_2_ chains coupled to a single cavity mode with ℏω_c_ = 2.98 eV and ℏη_c_ = 10^–2^ℏω_c_. The response is calculated either directly using the TDDFT + radiation reaction for all N dimers (black) or using the embedding radiation-reaction approach (red) where only a single dimer is calculated explicitly. Different combinations of light-matter coupling strengths  and particle numbers *N* are illustrated. Vertical gray-dashed lines serve as a guide to the eye for specific polaritonic resonances. Please refer to the text for further explanations. The excitations have been artificially broadened; see the SI for numerical details.

Using the embedding radiation-reaction ansatz is substantially cheaper than calculating the full dimer chain explicitly. While the cost of the latter outgrows quickly all available computational resources, our embedding approach retains the cost of a single-dimer calculation. This computational advantage opens up the possibility to investigate far more complex systems and will allow us to consider polaritonic chemistry truly from first principles.

During a chemical reaction, structural changes of the molecule will alter its vibrational spectrum. In between the reactant and product state, the molecule will spend time in intermediate configurations which will likely feature different vibrational energies and oscillator strengths. The closer this detuned vibration is to the collective polaritonic states, the stronger its contribution will outweigh the individual contribution of an ensemble molecule (see, e.g., SI Figure 1). While the reactant configuration will therefore play a negligible role in the polaritonic state, during the reaction our individual molecule might play a dominant role. Even if the number of dark states might be large, the bright states can play a dominant role in this scenario.

Let us illustrate this concept for a simple proton-tunneling model that is designed to represent ground-state reactions. A tilted double well potential *v*(*x*, *t*) = *x* × 10^−3^ − *x*^2^ × 1.25 × 10^−3^ + *x*^4^ × (1 + 0.4/[1 + *e*^−(*t* − 60*fs*)/10*fs*^]) × 10^−4^ (in a.u.) shown in Figure 2 is slightly deformed over a time frame of approximately *t*_p_ ≈ 0.1 ps. Such a deformation results in a weak excitation of the proton wave function and leads to a finite probability to overcome the barrier from the left to the right well. The amount of nuclear density that overcomes the barrier will be considered as an indication for the reactivity in our model. The role of the ensemble molecules is to modify the interaction between cavity mode and our impurity molecule; Figure 2b illustrates an exemplary *F*_*t*_^–1^*i*ω*G*(ω). Importantly, the collective coupling between cavity and ensemble has to be strong enough to induce beatings within the time frame of the reaction. This defines a minimal hybridization strength in **G**(ω) for a given reaction speed 1/Ω_Rabi_ ∼ *T*_Rabi_ < τ_reaction_—a quick reaction demands a large hybridization. We consider the ensemble in the following as a generic second species which is represented by a Drude–Lorentz model 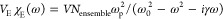


**Figure 2:**
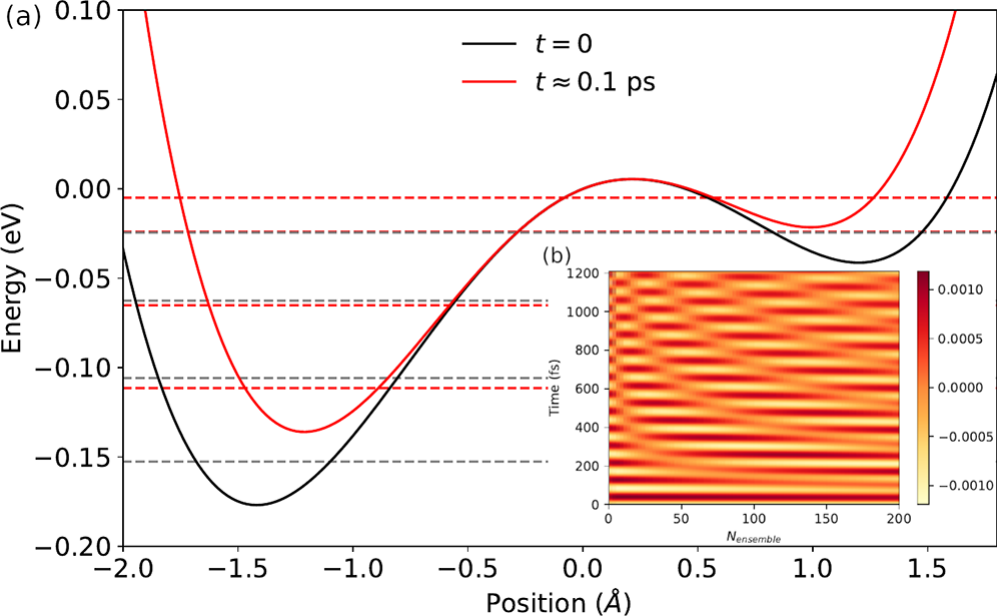
(a) Potential energy surface before (black) and after (red) the deformation. Dashed horizontal lines indicate eigenvalues of the respective static single-proton Schrödinger equation. (b) Time-domain convolution-argument *F*_*t*_^–1^*i*ω*G*(ω) for *g*_0_/ℏω_c_ = 0.0135, γ_e_ = 0.025ω_c_, η = 10^–4^ω_c_ (see text for elaboration). Only for sizable hybridization strength can we observe the Rabi period on the time scale of the reaction time.

Figure 3 (top) shows the absolute influence of resonant collective light-matter coupling on the accumulated proton tunneling for various combinations of fundamental coupling and ensemble size. The influence of the ensemble on the reaction depends largely on the single-particle coupling strength to our impurity molecule and is, even for *N*_ensemble_ = 0 (see top), already noticeably increased. Smaller coupling values (e.g., *g*_0_/ℏω_c_ = 0.0027) show the tendency to decrease this effect when increasing the ensemble size, as one would intuitively expect. Increasing the fundamental light-matter coupling does not only shift the resulting curve to smaller *N*_ensemble_ values but furthermore intensifies the overall trend. In the extreme case of *g*_0_/ℏω_c_ = 0.0135, we observe initially an increase in catalysis followed by an inhibiting effect for larger *N*_ensemble_. However, Figure 3 (bottom) illustrates that the proton-tunneling dynamic features a complex, alternating dependence on *N*_ensemble_. The latter is fluctuating between a 100% increase and almost full inhibition of proton-tunneling. Particularly striking is that the magnitude of the cavity influence remains comparable between *N*_ensemble_ = 0 and *N*_ensemble_ = 10^4^ or even increases in the case of *g*_0_/ℏω_c_ = 0.00054 (yellow). Even for *N*_ensemble_ > 10^8^ can fading resonances be observed. Figure 3 (bottom) would suggest that, depending on cavity parameters; selected resonances; and the mixture of reactant, product and solvent, a chemical reaction can be selectively catalyzed or inhibited. Both catalyzing^69^ and inhibiting effects have been observed in experiments using either vibrational^19,20^ or electronic strong coupling.^9,12^

**Figure 3:**
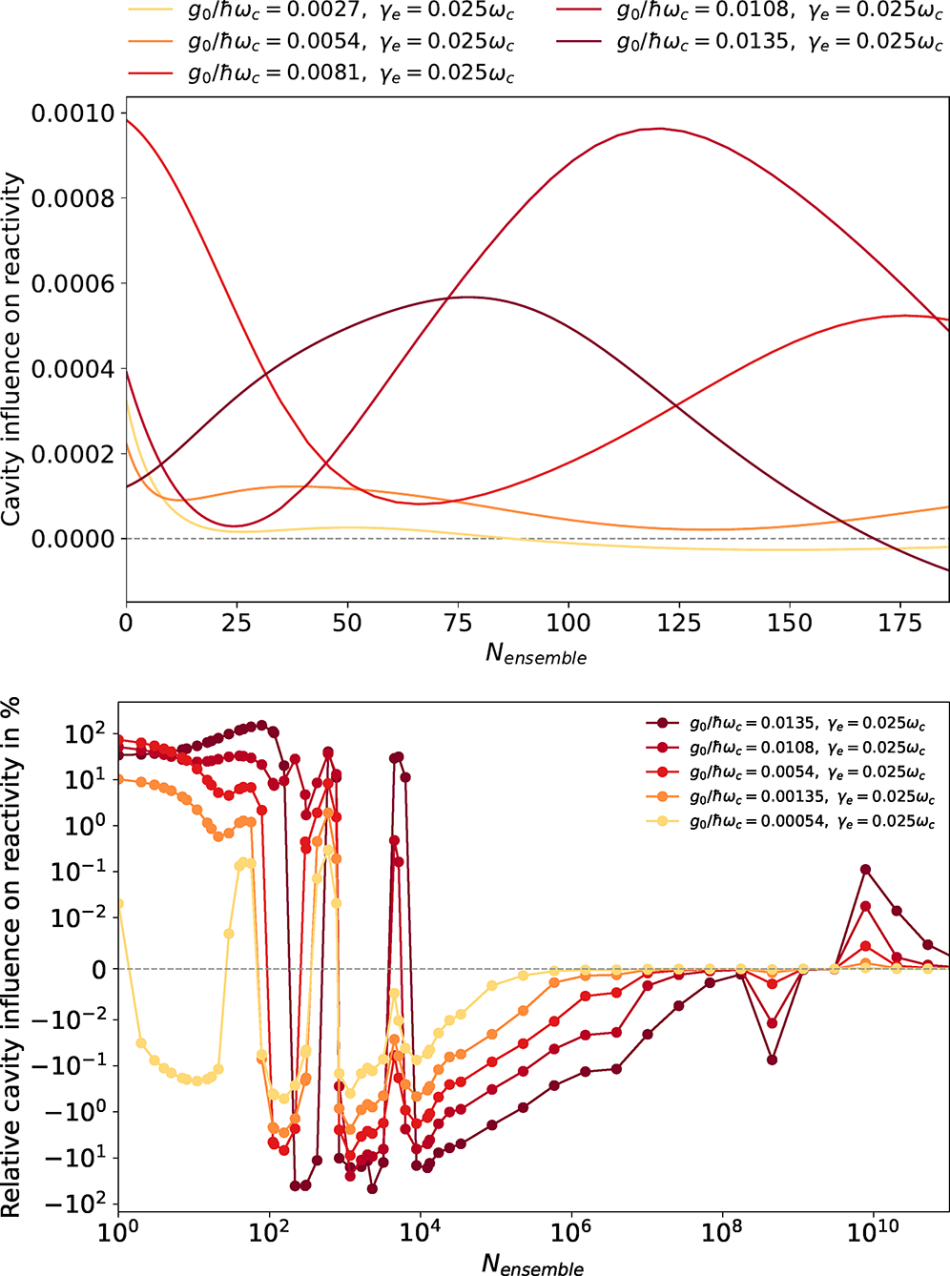
(Top) Absolute cavity influence on reactivity CR = ∫_0_^*T*^^d*T*^/*_T_*[1 – (∫_–∞_^0^*dxn*(*x*,*t*) – ∫_0_^∞^*dxn*(*x*,*t*))] – CR_*g*_0_=0_ for increasing the number of ensemble emitters. (Bottom) relative cavity influence CR/CR_*g*_0_=0_ in percent. The dependence of the cavity influence on the number of ensemble molecules *N*_ensemble_ is nontrivial and far from a simple 1/*N*_ensemble_ trend. Decoherences, for ensemble and cavity, are included but show no particularly striking effect as detailed in the SI. We use a Drude–Lorentz model with ℏω_p_ = 6.387 × 10^–4^ eV. The first excitations are aligned at *t* = 0 ℏω_c_ = 0.046721 eV = ℏω_E_ = ℏω_0–1_; the cavity decay rate is fixed to η = 10^–4^ω_c_. For large ensembles and coupling values, the light-matter hybridization reaches significant values which calls the semi-classical description into question. We start to enter this domain around *N*_ensemble_ = 10^4^ for the largest coupling.

Figure 4 (top) illustrates that the influence of collective light-matter coupling is sensitive to the energetic alignment between the ensemble, cavity mode, and reacting system. The associated charge-transfer dynamic is shown in Figure 4 (bottom). Such a complex behavior for our simple model demonstrates that the influence of collective strong coupling on chemical reactivity can be highly situational—a strong argument for the importance of *ab initio* approaches.

**Figure 4:**
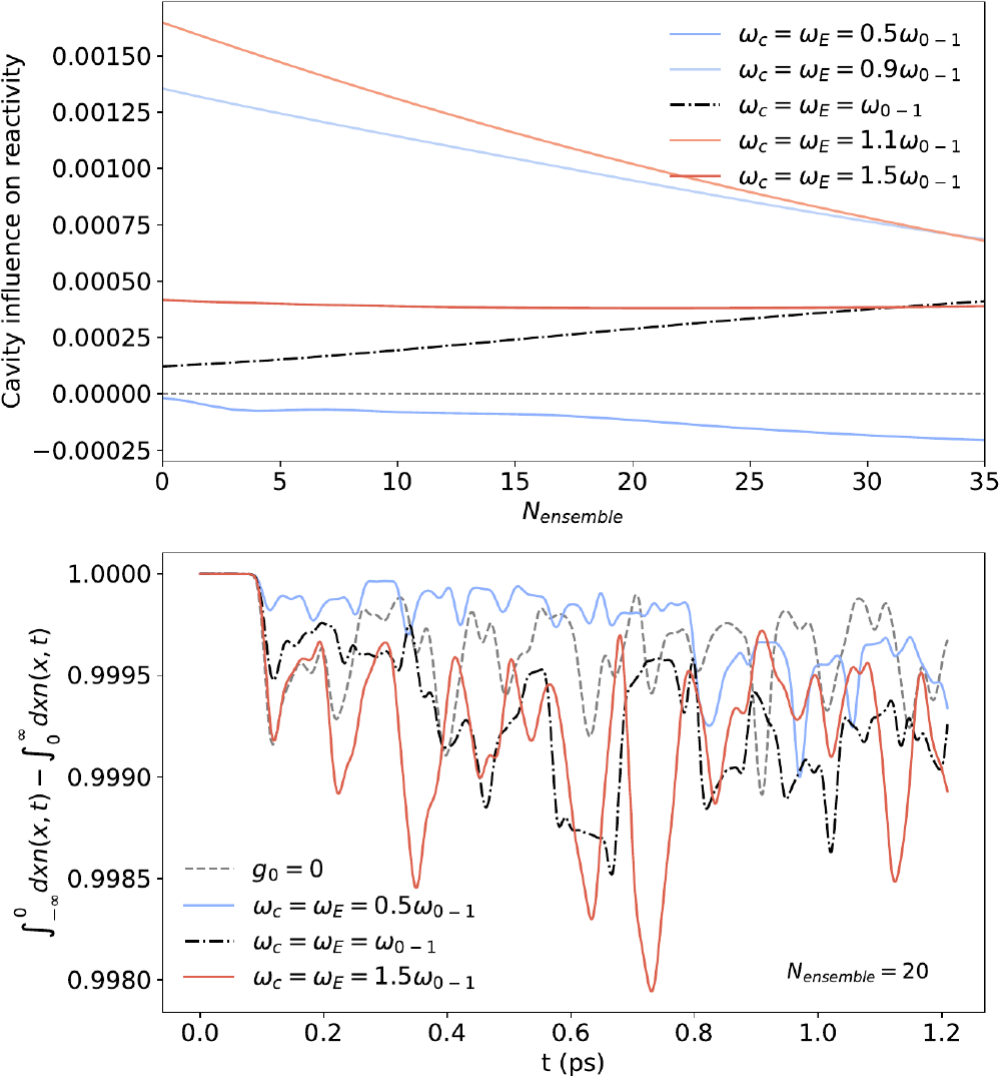
Top, absolute cavity influence on reactivity CR = ∫_0_^*T*^^d*T*^/*_T_*[1 − (∫_–∞_^0^*dxn*(*x*,*t*) – ∫_0_^∞^*dxn*(*x*,*t*))] – CR_*g*_0_=0_ for an increasing number of ensemble emitters for different energetic alignments between cavity/ensemble frequency and bare excitation frequency of our impurity molecule. Bottom, proton-tunneling (∫_–*∞*_^0^*dxn*(*x*,*t*) – ∫_0_^*∞*^*dxn*(*x*,*t*)) for *N*_ensemble_ = 20. The complex excitation structure leads to a nontrivial influence on the proton tunneling. In contrast to the other parameters, the smallest frequency tends to inhibit the proton-tunneling process. We fixed the cavity volume *V* in all calculations to the value of the resonant alignment. It is apparent from the behavior with increasing ensemble number *N*_ensemble_ that the specific effect of the cavity on the chemical reactivity is sensitive to the energetic alignment between cavity, ensemble, and reacting molecule as well as the hybridization between those systems.

Recall that the influence on the reactivity will depend not only on the energetic alignment between the reactant, product, solvent, and cavity but also its hybridization strength in relation to the reaction time. Identifying the specific influence of collective strong coupling on a chemical reaction remains an open and theoretically challenging problem. While the chosen proton-tunneling system is too simplistic to draw direct connections to the experiment, it demonstrates the feasibility of the introduced embedding radiation-reaction approach. Furthermore, it illustrates clearly that the cavity influence on chemical reactivity can persist even at a large *N*_ensemble_ and that the catalyzing and inhibiting effect could be present for the same chemical reaction. Recent work^22−24,70^ showed promising progress, and yet much remains to be investigated. A conclusive understanding will likely demand a collaborative effort involving experimental work, *ab initio* theory, and simplified models.

To conclude, by embedding an ensemble of molecules into the recently proposed radiation-reaction potential,^47^ we have been able to tackle the conundrum of a first-principles description of the material structure combined with the collective light-matter interaction involving a large number of emitters. The embedding ansatz is trivial to implement into existing time-dependent density-functional theory libraries as it adds a simple local potential. This allowed us to treat collective strong coupling from first-principles featuring an arbitrarily large number of emitters with marginal additional computational cost compared to ordinary real-time TDDFT calculations. Last, by tuning the cavity in the infrared regime, we demonstrated that the modification of proton-tunneling reactions via collective strong coupling can possess a nontrivial dependence on the number of ensemble molecules, featuring domains of a 100% increase in reactivity but also near full inhibition. Cavity losses decrease the effect of collective strong coupling on the tunneling process but have an overall negligible influence (see SI).

This work paves the way to tackling new regimes of theoretical chemistry, accounting for the self-consistent interaction between light with macroscopic systems while focusing on the microscopic dynamic of single subsystems—entirely from first principles. This allows us to relax previous simplifications to only single or few molecules with artificially increased coupling strength.^22,51,53,68^ Already our here illustrated simple proton-tunneling model exhibited nontrivial behavior with the number of emitters. We can expect that complex multistep chemical reactions will further complicate this trend—a strong argument for the need of first-principles techniques. The seamless combination of embedding radiation-reaction and quantum-electrodynamical density-functional theory, especially the recently developed local-density approximation,^56^ ensures that quantum-corrections of the light-matter interaction can be properly accounted for if necessary. Possible extensions include a consistent treatment of near-field effects that are relevant for solvation, plasmonic environments, and J/H aggregates. Equipped with this toolset, describing consistently the seminal experimental work in polaritonic chemistry^1−3^ moves within reach.

## Supporting Information Available

Simulation details, influence of decoherence, detuning and integration-time for the proton-tunneling reaction, connection between single-molecule and collective spectra, additional explanations, and limitations of the embedding radiation-reaction approach (PDF)
Transparent Peer Review report available (PDF)
