# Supplementary material for: Polaritonic Chemistry from First Principles via Embedding Radiation Reaction
Source: J Phys Chem Lett. 2022 Jul 22;13(30):6905–11. doi: 10.1021/acs.jpclett.2c01169 (PMC9358701; doi:10.1021/acs.jpclett.2c01169)
Supplement: Supplementary file 1 — jz2c01169_si_001.pdf [file jz2c01169_si_001.pdf]

Supporting Information: Polaritonic Chemistry from First Principles via Embedding Radiation-Reaction

Christian Schäfer*

Department of Microtechnology and Nanoscience, MC2, Chalmers University of Technology, 412 96 Göteborg, Sweden

E-mail: christian.schaefer.physics@gmail.com

Abstract

The supporting information includes additional information on the conception, application and limitation of the embedding radiation-reaction approach. Furthermore, we discuss how loss/decoherence affects the influence of collective strong coupling on the proton-tunneling reaction. Finally, we provide numerical details and discuss relevant aspects.

1 Illustration and Limitations

The moment we embed a macroscopic susceptibility into the dyadic Green tensor $\mathbf{G}_\perp$, the spectral response of the now dressed environment will change. If the excitation energies of $\chi_E(\omega)$ and $\mathbf{G}_0$ overlap and their interaction is non-zero, the dressed $\mathbf{G}_\perp$ will feature polaritonic excitations that are separated by the hybridization energy. Our individual molecule that hybridizes with those new quasi-particle excitations will be forced to oscillate at the excitation energies of the collective states. Figure S1 presents how an individual one-dimensional hydrogen contributes to the absorption of the collective state. The larger the number of ensemble emitters $N_{ensemble}$, the smaller its participation in the bright upper and lower polaritonic states. The spectral weight of the single molecules moves into an increasing number of dark states which are located at the original excitation energy. This bright/dark state behavior represents the hallmark of collective strong coupling and is at the heart of polaritonic chemistry[1,2]. By embedding the ensemble-dressed cavity into the electronic structure calculations, we obtain simultaneous access to the complex electronic dynamics and its interplay with macroscopic collective states.

A more complex bright-state dynamic appears if cavity, ensemble and explicit emitter are energetically detuned (see figure S2).

Cavity and ensemble will create the usual (large symmetric) splitting to which the detuned hydrogen contributes weakly. At larger $N_{ensemble}$, the bright ensemble states start to hybridize with the bare excitation of hydrogen, leading to upper, middle and lower polaritonic bright states. The hybridization strength between middle and lower polariton is dominated by the fundamental coupling strength between hydrogen and cavity. Close to the avoided crossing, the contribution of hydrogen to the hybridized state can be substantially larger than the contribution of an average ensemble emitter — at this point hydrogen and the bright ensemble state contribute equally to the full polaritonic states. This becomes apparent when comparing figure S1 to figure S2. Similar effects have been also ob-

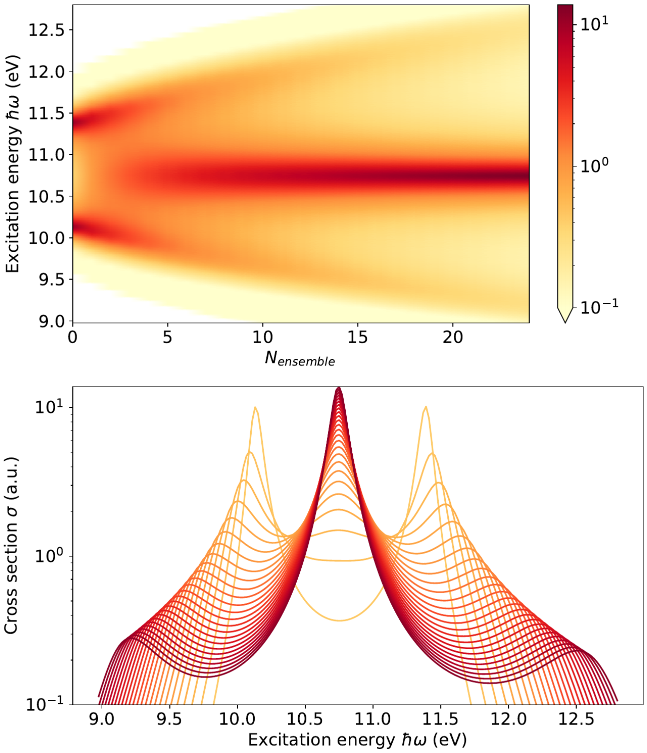

Figure S1: Photoabsorption cross-section $\sigma(\omega)$ of one-dimensional hydrogen coupled with strength $g_0/\hbar\omega_c = 0.0563$ to a single cavity mode in resonance to $\hbar\omega_{eg} = 10.746$ eV with loss $\hbar\eta = 10^{-2}\hbar\omega_{eg}$ which is dressed by $N_{ensemble}$ ensemble emitters. The ensemble response was represented by a Drude-Lorentz model $V_E\chi_E(\omega) = VN_{ensemble}\cdot\omega_p^2/(\omega_0^2-\omega^2-i\gamma\omega)$, $\hbar\omega_p = 3\sqrt{10}/200\ eV$, $\gamma = 0.1\cdot\omega_0$, $\omega_0 = \omega_c$.

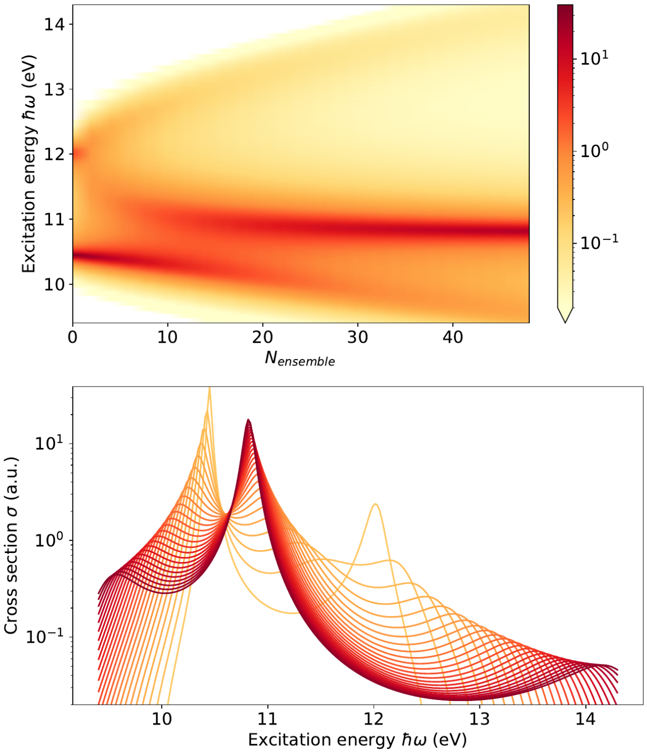

Figure S2: Photoabsorption cross-section $\sigma(\omega)$ of one-dimensional hydrogen coupled coupled with strength $g_0/\hbar\omega_c = 0.0539$ to a single cavity mode which is detuned at $\hbar\omega_c = 11.7$ eV with loss $\hbar\eta = 10^{-2}\hbar\omega_{eg}$ and dressed by $N_{ensemble}$ ensemble emitters. The ensemble response was represented by a Drude-Lorentz model $V_E\chi_E(\omega) = VN_{ensemble}\cdot\omega_p^2/(\omega_0^2-\omega^2-i\gamma\omega)$, $\hbar\omega_p = 3\sqrt{10}/200\ eV$, $\gamma = 0.1\cdot\omega_0$, $\omega_0 = \omega_c$, $V_E/V = 10^{-5}$.

served with quantum-electrodynamical density-functional theory[3]. The following Sec. 1.1 illustrates how the embedding radiation-reaction approach and the here discussed observations can be understood from the perspective of simple quantum optical models.

An important limitation of this simplified approach rests in the self-consistency of the embedding procedure and the strength of any external perturbation. We made two assumptions, first, the ensemble can be described as linear medium, i.e., external fields are weak. While we could apply the radiation-reaction potential without issue for the full ensemble at once to treat e.g. high-harmonic generation[4], i.e., $\varepsilon_r = 1$, $\mathbf{j} = \sum_{i=1}^{N_E+1}\mathbf{j}_i$ and $\mathbf{G} = \mathbf{G}_0$, the resulting computational cost would be prohibitive for larger systems. The second, the cavity field exerts only weak forces on the ensemble. This limit is fulfilled in the weak and strong coupling regimes for light-matter interaction, i.e., cavity and ensemble can hybridize but the underlying matter-response experiences only minor changes. When reaching the ultra-strong coupling regime between light and matter, the underlying electronic or nuclear excitation structure will be modified considerably[5–9]. We can account for those change by e.g. using the QEDFT local-density approximation proposed in[7] which is fully compatible with the here proposed radiation-reaction potential. We demonstrate such a combination in Sec. 2 and show that, for the here selected parameter regime, the QEDFT corrections are negligi-

ble. Furthermore, we can self-consistently update all components if extensions into stronger coupling regimes are envisioned. Imagine we couple a small set of molecules very strongly to the cavity but embed all but one into the Green tensor, substantial light-matter interaction will change the response of each molecule, which in turn affects the dressed Green tensor — a self-consistent cycle emerges in which χ, $\mathbf{j}$ affect $\mathbf{G}$ and the latter modifies χ, $\mathbf{j}$. Such a generalized embedding scheme, which appears particularly appealing in the linear response regime, and its combination with the local-density approximation will be the subject of future work. The real-time propagation employed in the present work provides natural access out-of-equilibrium molecular dynamics and conveniently integrates cavity and ensemble lifetimes into the microscopic description.

Collective strong coupling experiments are safely within the strong coupling regime and typically performed in the 'dark', i.e., without or with only weak external drive[10–12]. The conditions of the illustrated approach are therefore satisfied. Most importantly, both limitations are only mildly limiting the dynamic of the explicitly treated molecule. It is entirely within the limits of the approximation if our explicit molecule undergoes for instance a charge reorganization or a chemical reaction — both considered to be of major interest in QED chemistry and QED material design.

1.1 Illustration of the embedding at the Hopfield model

We illustrate here how the embedding procedure can be understood from the perspective of the quantum-optical Hopfield model for hybridizing modes. This widely used and strongly simplified model assumes the dipolar approximation, the rotating-wave approximation and emitters are approximated as bare 2-level systems. Expanding the corresponding Hamiltonian $\hat{H}_{sH} = \omega_c \hat{a}^\dagger \hat{a} + \sum_i^N \omega_i \sigma_{z,i} + \sum_i^{N+1} g_i(\hat{a}\sigma_i^+ + \hat{a}^\dagger \sigma_i^-)$ into the excitation basis leads to a matrix equation that is commonly fitted in order to analyze experimental data.

The interaction between an ensemble of collectively interacting modes, describing a large set of ensemble molecules, a selected single molecule, and a cavity mode, will be given under the above approximations by the simplified Hamiltonian-matrix

$$\begin{pmatrix} \omega_c & g & \cdots & g_m \\ g & \omega_{E,1} & 0 & 0 \\ g & 0 & \ddots & 0 \\ g_m & \cdots & 0 & \omega_m \end{pmatrix}$$

for which the N ensemble molecules posses the identical excitation energy $\omega_{E,1} = \omega_{E,N}$ and light-matter coupling g. In this case, arrowhead structure allows us to remove all those degenerate eigenvalues from the matrix by separating the problem into symmetric (also known as bright) and antisymmetric (also known as dark) sub-blocks.

For example, the simplest case of this block-diagonalization is given for 2 emitters coupled to one mode. The Hamiltonian matrix

$$\begin{pmatrix} \omega_c & g & g \\ g & \omega_E & 0 \\ g & 0 & \omega_E \end{pmatrix}$$

can be separated via $\mathbf{S}^T \mathbf{H} \mathbf{S}$ where

$$\mathbf{S} = \begin{pmatrix} 1 & 0 & 0 \\ 0 & 1/\sqrt{2} & -1/\sqrt{2} \\ 0 & 1/\sqrt{2} & 1/\sqrt{2} \end{pmatrix}$$

into block-structure

$$\begin{pmatrix} \omega_c & \sqrt{2}g & 0 \\ \sqrt{2}g & \omega_E & 0 \\ 0 & 0 & \omega_E \end{pmatrix}$$

where the cavity couples now only to the symmetric/bright state with amplified strength $\sqrt{2}g$ while the antisymmetric/dark state is decoupled from the cavity. The same can be achieved by noticing that for identical coupling and excitation energy the sum of excitation operators $\sum_i^{N+1} g_i(\hat{a}\sigma_i^+ + \hat{a}^\dagger \sigma_i^-)$ can be simplified to collective excitation operators $S^\pm = \sum_i^N \sigma_i^\pm$, also called Dicke operators.

We reduce ourselves now to the bright-state

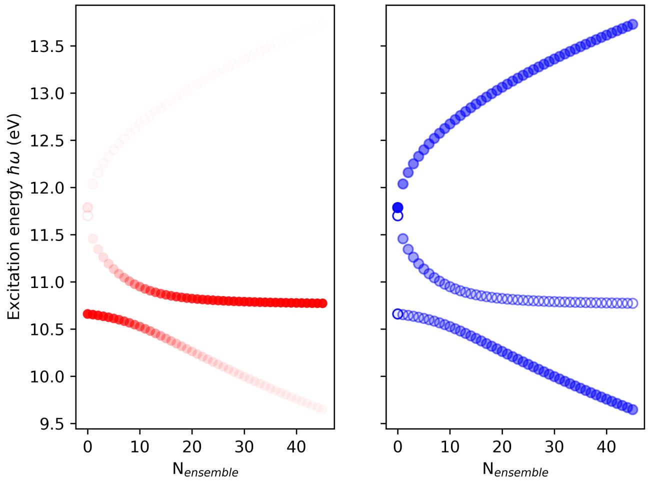

Figure S3: Simple Hopfield model for the interaction of an ensemble of identical molecules coupled to a single cavity mode which again is coupled to a single detuned molecule. Left, contribution of the single molecule to the collective states. Right, photonic contribution. Parameters $\hbar\omega_c = \hbar\omega_E = 11.7\ eV$, $\hbar\omega_m = 10.746\ eV$, $g = g_m = 0.011$.

sub-manifold described by

$$\begin{pmatrix} \omega_c & g\sqrt{N} & g_m \\ g\sqrt{N} & \omega_E & 0 \\ g_m & 0 & \omega_m \end{pmatrix} = \begin{pmatrix} \omega_E & g\sqrt{N} & 0 \\ g\sqrt{N} & \omega_c & g_m \\ 0 & g_m & \omega_m \end{pmatrix}$$

with the excitation structure illustrated in figure S3.

At the moment, the ensemble and the single molecule couple still to the bare mode. Let us assume $\omega_c = \omega_E = 1$ for simplicity. The upper-left 2-by-2 block possesses the eigenvalues $\omega_\pm = 1 \pm g\sqrt{N}$ and eigenvectors $\mathbf{v}_\pm^T = (\pm 1/\sqrt{2}, 1/\sqrt{2})$. The transformation $\mathbf{S}^{-1}\mathbf{H}\mathbf{S}$ with $\mathbf{S} = ((\mathbf{v}_1, 0)^T, (\mathbf{v}_2, 0)^T, (0,0,1)^T)$ leads to

$$\begin{pmatrix} \omega_+ & 0 & g_m/\sqrt{2} \\ 0 & \omega_- & g_m/\sqrt{2} \\ g_m/\sqrt{2} & g_m/\sqrt{2} & \omega_m \end{pmatrix} .$$

and the single molecule couples now to the collective bright states (upper and lower polariton $\omega_\pm$). The embedding procedure via the ensemble-dressed Green tensor is conceptually similar. The dressed $\mathbf{G}$ has poles at the bright ensemble+environment eigenvalues with adjusted strength, dark states are not present as they do not provide any spectral intensity from the perspective of the cavity. The microscopic material described via the Schrödinger equation is then affected by radiation-reaction forces which are maximal at the polaritonic resonances. The here introduced embedding scheme can be seen as a generalization of the simplified Hopfield model to realistic systems. This generalization allows us to investigate time-dependent local phenomena for realistic systems, i.e., it provides access to chemical reactions, charge-transfer processes and many other features while retaining the full collective interaction. Clearly, the underlying assumptions that the majority of the ensemble is perfectly identical and responds only weak/linear are strong limitations to the embedding approach. More complex systems will demand a more sophisticated separation of the different embedded species, the specific path chosen here is designed to address the conditions for collective strong-coupling in polaritonic chemistry.

2 Ultra-strong coupling effects - combining radiation-reaction with QEDFT-LDA

Every light-matter coupling strength greater than zero will alter the electronic and nuclear structure, reorganizing charge and breaking symmetries[5]. Typically, those effects are referred to as ultra-strong coupling features and their second-order scaling $\mathcal{O}(g_0^2)$, compared to the $\mathcal{O}(g_0^1)$ scaling of polariton hybridization, renders them of minor relevance for the vast majority of experimental realizations. Exceptions are located in the domain of plasmonics[13] and circuit-QED[14], the latter allows even coupling strengths on the order of the excitation energy $g/\hbar\omega \approx 1$. Quantum-electrodynamical density-functional theory (QEDFT) allows to consider such quantum-corrections also for realistic systems. We employ here the recently proposed photon-exchange local-density approximation (pxLDA)[7] in combination with the radiation-reaction potential, an easy task as

the latter accounts for the classical Maxwell-components and the pxLDA potential provides access to quantum-fluctuations.

Figure S4 (top) exemplifies the renormalization of the electronic ground-state density for the in figure S1 performed calculation with $N_{ensemble} = 0$. The effect is minute for the chosen couplings which transfers into a negligible spectral shift (figure S4 (bottom), red dashed) based on this change of the initial-state. Usage of the adiabatic pxLDA potential during the time-propagation (magenta dashed-dotted) provides a very weak blue-shift. Consequently, the pxLDA is overall of minor relevance in our calculations and we will omit quantum-effects in the rest of this work. Note however, that such a conclusion is system and parameter specific and adding pxLDA or comparable corrections should be considered for each realization individually.

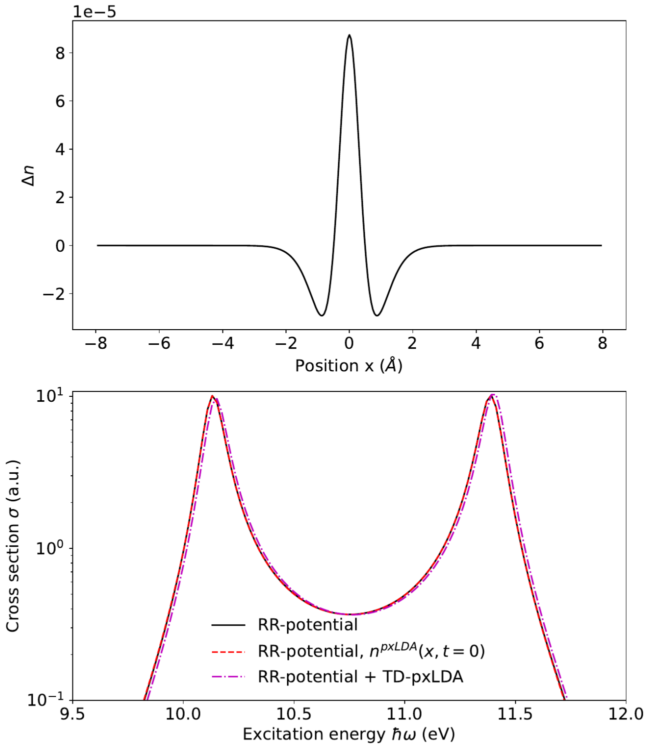

Figure S4: (Top) Ground-state electronic density renormalization $\Delta n(x) = n^{g_0/\hbar\omega=0.0563}(x) - n^{g_0=0}(x)$ using the pxLDA potential[7] for the system investigated in figure S1 with $N_{ensemble} = 0$. (Bottom) Photoabsorption cross-section $\sigma(\omega)$ excluding the adiabatic pxLDA functional (black solid), accounting only for the quantum-effects on the initial-state density (red dashed), and accounting for quantum-corrections also during the time-propagation with the help of the adiabatic pxLDA approximation (magenta dashed-dotted).

3 Collective state response from single-molecule response

The here demonstrated embedding approach gives access to the dynamic of a single molecule of a collectively coupled ensemble, paving a way towards an *ab initio* descriptions of polaritonic chemistry. Nevertheless, it is entirely possible to reconstruct the collective state response. The poles at which the embedded molecule oscillates coincides with the collective state resonances, the collective state can be reconstructed in two ways.

Option 1 is based on our knowledge about the system of identical emitters (obtained e.g. via the intuition obtained from the Tavis-Cummings or Hopfield model): The ratio between bright and dark states in the polarizability per molecule scales as $\frac{2}{N}$ for both bright states together vs $\frac{(N-1)^2}{N}$ for dark states (always 2 bright states but N-1 dark states, 1/N is the normalization per molecule). Since N identical molecules oscillate, a factor N is added that cancels the normalization. Multiplication of the bright states of our single (embedded) molecule by $2 + (N-1)^2$ provides therefore a very good estimate of how strong the bright states of the collective state will be. This simple connection, emerging purely from the re-scaling of spectral density, provides already quite accurate results as shown in figure S5 (top).

The dark states do not exist in the direct (black) calculation due to destructive interference, i.e., we would in principle need to cut those states from the spectrum. Clearly, if the embedding environment becomes more complex, such an intuitive and model-inspired explanation becomes no longer reliable. Option 1 neglects the fact that for a more general system,

the actually measured spectrum due to absorption/transmission will of course not only consist of the sum of simply re-scaled dipoles but must account for the correct electromagnetic emission. What we measure is not a dipole moment but the radiated/absorbed electric field.

Option 2 provides a more rigorous approach: The radiated field is given by

$$\begin{aligned} \mathbf{E}_{\perp,r}(\omega) &= i\mu_0\omega\mathbf{G}_{\perp}(\omega)\cdot(-i\omega)\mathbf{R}(\omega) \\ &= \mu_0\omega^2\mathbf{G}_{\perp}(\omega)\cdot\boldsymbol{\alpha}(\omega)\cdot\delta\mathbf{E}\ . \end{aligned} \quad (1)$$

The multiplication with the full Green's function (including N times the single molecule $\boldsymbol{\alpha}$) weights correctly bright states and eliminates dark states. Clean spectra will demand a high frequency resolution and since the cavity is only weakly decaying in our example (strong coupling condition), very long propagation times would be necessary. This is circumvented in figure 1 by artificial broadening. In order to take the above multiplications with sufficient accuracy and consistent norm, much higher resolution would be needed. Nevertheless, by again artificially broadening the dipole response we can provide a proof-of-principle calculation shown in figure S5 (bottom). As detailed in the numerical section, the frequency resolution for $\mathbf{G}_{\perp}$ is increased and well converged in our calculations but for the multiplication it is necessary to project back on the corse grid on which $\mathbf{R}(\omega)$ is given. The resulting spectrum of the radiated field are qualitatively close, the high of of the black excitations is sensitive to the low resolution of the direct calculations and the artificial broadening. Overall, the dark states are eliminated and upper and lower polariton are recovered (besides deviations due to the broadening). Quantitative comparisons would demand much larger losses for the cavity or longer propagation. Longer propagation times are easily accessible for the embedding approach but are costly for the direct calculation.

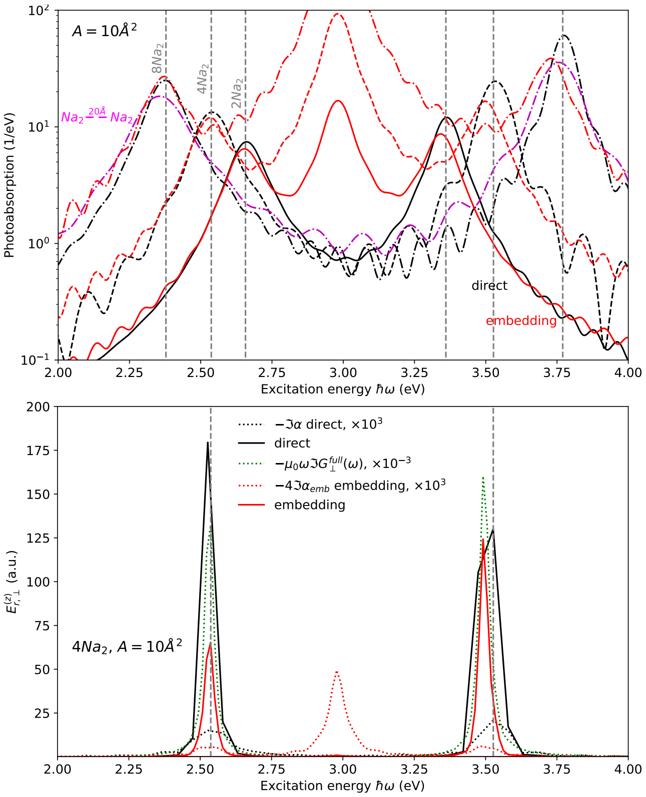

Figure S5: (Top) Photoabsorption of Na_2 chains coupled to a single cavity mode, same set-up as figure 1. The response is calculated either directly using TDDFT+radiation-reaction for all N dimers (black) or using the embedding radiation-reaction approach (red) where only a single dimer is calculated explicitly and then rescaled by $2 + (N-1)^2$ to reach the collective bright-state absorption. The dark states around 3 eV disappear due to their anti-symmetric super-position. Artificially broadening in combination with a mapping on a higher frequency resolution has been used).
(Bottom) Calculated $E_{\perp,r}^{(z)}$ with the direct use of $\mathbf{R}(\omega)$ (artificial broadening but no increase in resolution) for the cross section and its weighting according to eq. (1). A consistent comparison between direct collective-state response and the collective response obtained via the embedding approach would demand longer propagation and a comparison for the radiated/fields. The kick-strength is 10^{-5}, propagation times are 75 fs (direct) and 340 fs (embedding).

4 Influence of loss, speed of perturbation, detuning and integration time on the proton-tunneling reaction

Figure S6 shows the with figure 3 of the main text consistent calculations compared to calculations with quicker deformation (5 fs instead of 0.1 ps) or larger ensemble-loss (bottom). Quicker deformation of the potential deposits more energy in the system, resulting in overall larger reactivity and larger absolute cavity influence. Larger loss-rates smooth-out or flatten the cavity-influence but remain qualitatively similar to the values chosen in the main text.

Figure S7 presents $|\Im G(\omega)|$ consistent with figure 2 (b) of the main text.

Figure S8 is comparable to figure 4 in the main manuscript but instead of detuning ensemble and cavity, we only detune the cavity here. The differences are small, most likely due to the large energetic spacing and the linewidth of the ensemble emitters.

Figure S9 shows the relative cavity influence for different integration times $T = \{0.8, 0.9, 1.0\} \cdot T_{\text{main paper}}$. The results depend only marginally on T, visible only for small $N_{ensemble}$.

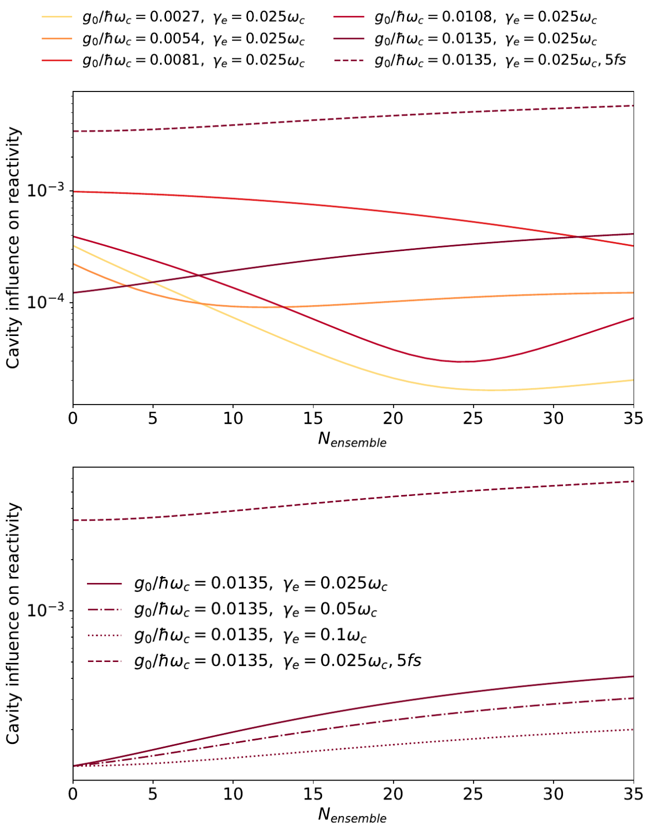

Figure S6: Cavity influence on reactivity $CR = \int_0^T \frac{dt}{T}[1 - (\int_{-\infty}^0 dxn(x,t) - \int_0^\infty dxn(x,t))] - CR(g_0 = 0)$ for increasing number of ensemble emitters. Top, CR for varying light-matter coupling-strength between single perturbed molecule and cavity (the first excitations are aligned at t=0 $\hbar\omega_c = 0.046721\ eV = \hbar\omega_E = \hbar\omega_{0-1}$, cavity decay rate fixed to $\eta = 10^{-4}\omega_c$). The dependence of the cavity influence on the number of ensemble molecules $N_{ensemble}$ is non-trivial, i.e., does not simply decrease with $1/N$ as often believed. A critical feature is the appearance of beating signatures discussed in figure 2 of the main text. Shorter perturbation times (dashed, $v(x,t) = x \cdot 10^{-3} - x^2 \cdot 1.25 \cdot 10^{-3} + x^4 \cdot (1 + 0.4/[1 + e^{-(t-5fs.)/fs}]) \cdot 10^{-4}\ H$) inject more energy into the system, leading to a larger reactivity and an enhanced cavity influence. Bottom, CR for different ensemble emitter-linewidths (solid, dashed-dotted, dotted) as well as for a shorter deformation period (dashed). Quicker ensemble decoherence leads to broad spectral response which tends to flatten the influence of collective strong coupling on the proton tunneling.

5 Numerical details

The radiation-reaction potential and the according convolution have been implemented into a small python3 library and the open-source code GPAW. The time-evolution can be efficiently calculated by finite-difference approximations. The implementation has been checked for consistency against pre-existing Maxwell-QEDFT calculations[7].

Close to lossless electromagnetic environments feature sharp peaks in the spectral response, e.g. a one-dimensional Fabry-Pérot cavity will be described by a set of harmonic oscillators. In order to allow a reliable numeri-

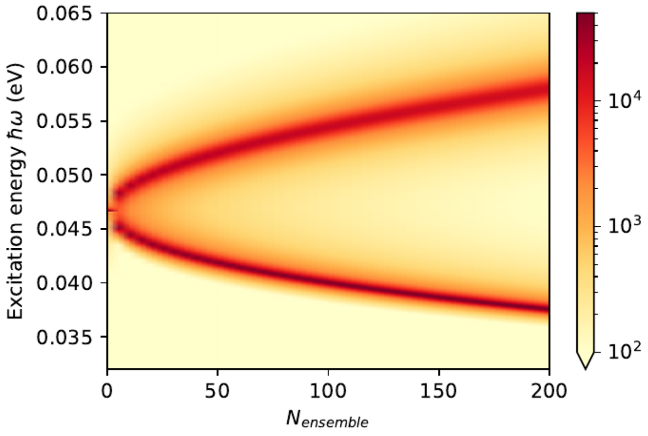

Figure S7: Excitation spectrum $|\Im G(\omega)|$ of ensemble+cavity for $g_0/\hbar\omega_c = 0.0135$, $\gamma_e = 0.025\omega_c$, $\eta = 10^{-4}\omega_c$.

cal description of Fabry-Pérot cavities, we calculated the Green tensor via

$$g_0(\omega) = \frac{2}{c^2 V}\mathcal{F}_t e^{-\eta t}\sum_{\mathbf{k}} \omega_{\mathbf{k}}^{-1}\sin(\omega_{\mathbf{k}} t)\ .$$

An apparent but critical feature emerges when obtaining $g(\omega)$. The frequency resolution has to be sufficient to properly resolve g_0 and χ, both can become sharp for small losses (= high Q values). It is therefore important to converge the spectral representation of $g(\omega)$, for instance by artificially extending the time put into the construction of $g(\omega)$. Alternatively, a variable resolution along the lines of wavelets could be used. A good indication of under-convergence for the Fabry-Pérot example is when the imaginary part of $g_0(\omega)$ (Lorentzian shape) changes sign around a resonance. In this work, we increased the frequency resolution, compared to the natural resolution given by the propagation-time, by a factor of 2000 for all reactivity calculations. The Green tensor $G(\omega)$ and $G(t)$ have been plotted with a 150 times higher resolution as they are visually identical from this point on and have only been used for visualization. Note, that this resolution is not sufficient to obtain an accurate description of the rather small and thus highly sensitive proton-tunneling process.

Figure S10 illustrates the possible consequences of choosing a too small frequency resolution. Compared are two runs with different artificially extended times (factor 1800 and

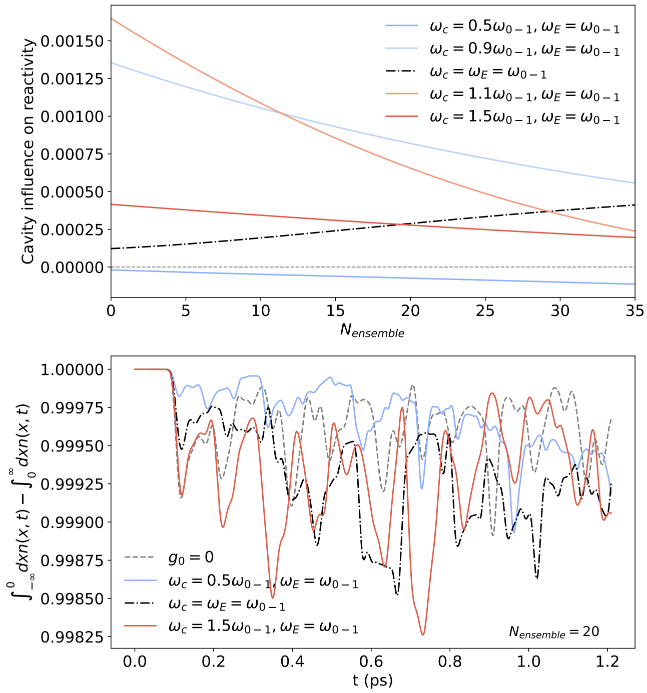

Figure S8: Top, absolute cavity influence on reactivity $CR = \int_0^T \frac{dt}{T}[1 - (\int_{-\infty}^0 dxn(x,t) - \int_0^\infty dxn(x,t))] - CR_{g_0=0}$ for increasing number of ensemble emitters for different energetic alignments between cavity/ensemble frequency and bare excitation frequency of our impurity molecule. The difference to figure 4 in the main manuscript is that the ensemble emitters are kept fixed at their excitation energy and only the cavity frequency is detuned. Since cavity and emitters feature a large loss-rate, detuning only the cavity seems to lead to slightly smaller effects compared to detuning ensemble and cavity at the same time. Bottom, proton-tunneling $\int_{-\infty}^0 dxn(x,t) - \int_0^\infty dxn(x,t))$ for $N_{ensemble}$. The complex excitation-structure leads to a non-trivial influence on the proton-tunneling. In contrast to the other parameters, the smallest frequency tends to inhibit the proton-tunneling process. We fixed the cavity volume V in all calculations to the value of the resonant alignment.

2000) for obtaining $g_0(\omega)$. While they are overall in good agreement, the (only slightly) under-converged run with factor 1800 misses the peak between 10^8 and 10^9. Furthermore, we can observe a tiny artificial offset for 1800. The calculation with factor 2000 (chosen in figure 3 of the main paper) are well converged. It should

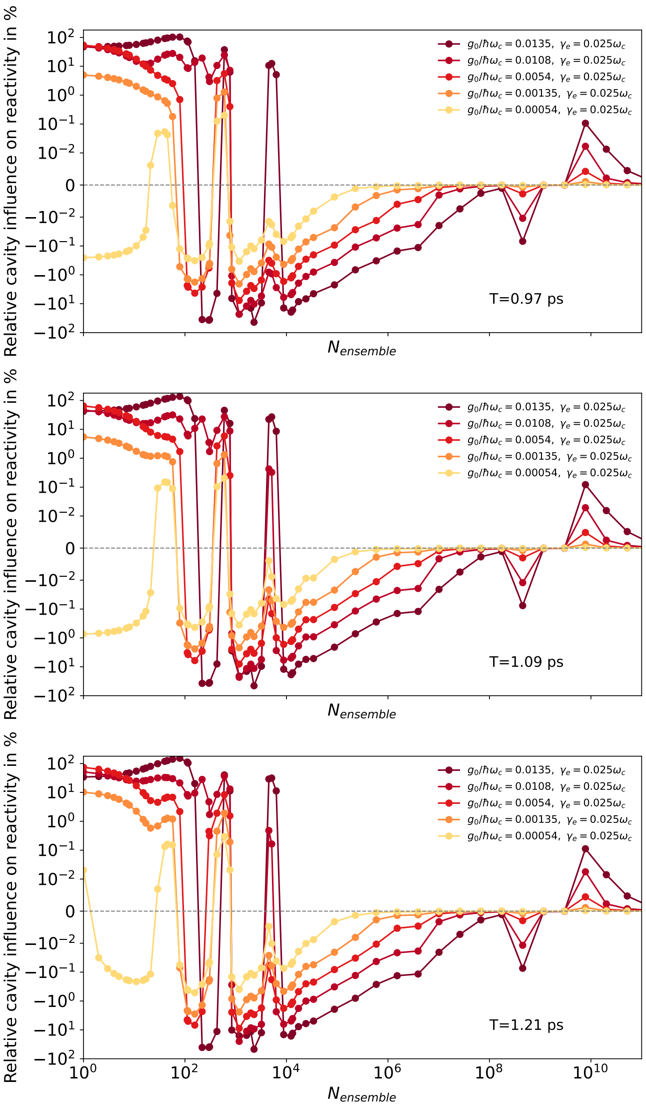

Figure S9: Relative cavity influence $CR/CR_{g_0=0}$ with $CR = \int_0^T \frac{dt}{T}[1 - (\int_{-\infty}^0 dxn(x,t) - \int_0^\infty dxn(x,t))] - CR_{g_0=0}$ for different integration (reaction) times. The prediction depends only marginally on the finite integration time, visible differences can be observed for small $N_{ensemble}$. This trend is expected as increasing $N_{ensemble}$ results in a larger Ω_{Rabi} and thus more oscillations within a fixed time. The influence of collective strong coupling on the proton-tunneling reaction converges therefore quickly for large $N_{ensemble}$.

be noted that the Rabi-splitting reaches sizable values for $g_0/\hbar\omega_c = 0.0135$ and $N_{ensemble} > 10^4$ which limits the reliability of the semi-classical approximation for the light-matter interaction.

Linear response calculations have been performed with a 10 times higher resolution as

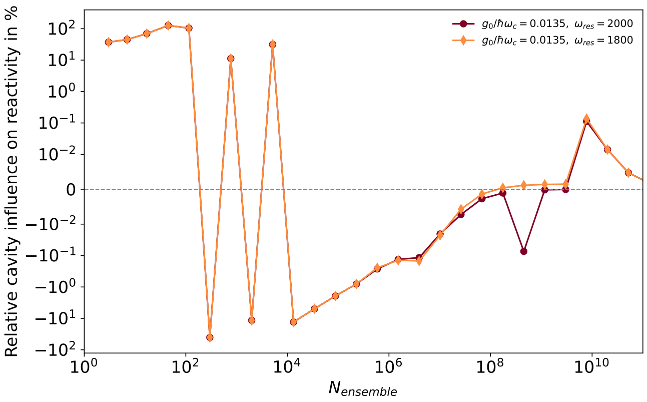

Figure S10: Relative cavity influence $CR/CR_{g_0=0}$ with $CR = \int_0^T \frac{dt}{T}[1 - (\int_{-\infty}^0 dxn(x,t) - \int_0^\infty dxn(x,t))] - CR_{g_0=0}$ – same parameters as in figure 3 (main manuscript, bottom). Even slight under-convergence can lead for large $N_{ensemble}$ to noticeable deviations. See text for more details.

they feature already from the start a higher resolution. All GPAW calculations used a 1000 times higher resolution. The time-stepping remained unchanged such that the $\mathcal{F}_t^{-1}\mathbf{G}(\omega)$ and the TDDFT propagation are consistent on the same time-interval. The additional computational cost can become noticeable if the resolution is pushed to extreme values as the FFT becomes then time-consuming. For most applications the cost will remain small or even negligible as FFTs are well optimized and $\mathbf{G}$ is constructed only once before the actual TDDFT calculation.

The subsequent real-time object $\mathcal{F}_t^{-1} i\omega\mathbf{G}(\omega)$ can be contaminated with overtones close to $t = 0^+$, the explicit variant $\mathcal{F}_t^{-1} i\omega\mathbf{G}(\omega) = -\partial_t\mathcal{F}_t^{-1}\mathbf{G}(\omega)$ is preferred. Attention should be devoted to the different possible definitions of the Fourier transformation and their discrete counterparts. The macroscopic χ_E is represented either from first-principles or via a Drude-Lorentz model with parameters as indicated in the captions. The presented cross sections in figure S1 and S2 feature artificial sign-flips towards lower and higher energies (seen from the resonances) as consequence of the finite time-interval. The artificial features were ignored here as they appear at small cross sec-

tion value and do not affect the conclusions. Stronger damping or longer propagation would remove them if deemed necessary.

Figure S1 and S2 have been calculated on a grid with 301 grid-points, 0.1 a_0 spacing and 4th-order finite-difference. The linear-response spectrum has been obtained by perturbing the electronic system with a Lorentzian-shaped delta-kick $v(x,t) = -10^{-4}\frac{1}{\pi}\frac{10^{-2}}{(t-1)^2+10^{-4}}x$ (in a.u.) and time-propagation for 800001 time-steps with $\Delta t = 0.01$ a.u. and 4th-order Runge-Kutta.

Figure 1 of the main text was obtained in multiple steps as described in the main text. The polarizability of a single ensemble sodium dimer was calculated by real-time propagation via $\alpha_{zz}(\omega) = R_z(\omega)/(2\pi K)$ ($K = 10^{-5}$ being the kick-strength) using the LCAO routine of GPAW[15,16] including the radiation-reaction potential[4] with a cross-sectional area $A = 10^2$ Å^2. The subsequently obtained spectra using the embedding radiation-reaction calculations used the default real-time to spectrum tool of GPAW with a Lorentzian broadening of 0.05 eV (0.08 eV for $8Na_2$ with 20 Å distance). All calculations used a spacing of 0.3 Å, a box of size 24+chain-length$\times 24 \times 24$ Å^3, the atomic p-valence dz basis and the LDA functional. The sodium dimers have a fixed bonddistance of 1.104 Å.

All reactivity calculations have been performed on a grid with 301 grid-points, 0.04 a_0 spacing and 4th-order finite-difference. The particle-mass was adjusted to the proton-mass. The real-time dynamics involved the deformation of the potential energy surface as specified in the main text with 100001 time-steps with $\Delta t = 0.5$ a.u. and 4th-order Runge-Kutta.

References

(1) Ebbesen, T. W. Hybrid Light–Matter States in a Molecular and Material Science Perspective. *Acc. Chem. Res.* **2016**, *49*, 2403–2412.

(2) Yuen-Zhou, J.; Menon, V. M. Polariton chemistry: Thinking inside the (photon) box. *Proc. Natl. Acad. Sci. U.S.A.* **2019**, *116*, 5214–5216.

(3) Sidler, D.; Schäfer, C.; Ruggenthaler, M.; Rubio, A. Polaritonic chemistry: Collective strong coupling implies strong local modification of chemical properties. *The journal of physical chemistry letters* **2020**, *12*, 508–516.

(4) Schäfer, C.; Johansson, G. Shortcut to Self-Consistent Light-Matter Interaction and Realistic Spectra from First Principles. *Phys. Rev. Lett.* **2022**, *128*, 156402.

(5) Schäfer, C.; Ruggenthaler, M.; Rubio, A. Ab initio nonrelativistic quantum electrodynamics: Bridging quantum chemistry and quantum optics from weak to strong coupling. *Phys. Rev. A* **2018**, *98*, 043801.

(6) Haugland, T. S.; Schäfer, C.; Ronca, E.; Rubio, A.; Koch, H. Intermolecular interactions in optical cavities: An ab initio QED study. *J. Chem. Phys.* **2021**, *154*, 094113.

(7) Schäfer, C.; Buchholz, F.; Penz, M.; Ruggenthaler, M.; Rubio, A. Making ab initio QED functional (s): Nonperturbative and photon-free effective frameworks for strong light–matter coupling. *Proceedings of the National Academy of Sciences* **2021**, *118*.

(8) Flick, J.; Schäfer, C.; Ruggenthaler, M.; Appel, H.; Rubio, A. Ab Initio Optimized Effective Potentials for Real Molecules in Optical Cavities: Photon Contributions to the Molecular Ground State. *ACS Photonics* **2018**, *5*, 992–1005.

(9) Latini, S.; Shin, D.; Sato, S. A.; Schäfer, C.; De Giovannini, U.; Hübener, H.; Rubio, A. The ferroelectric photo ground state of $SrTiO_3$: Cavity materials engineering. *Proceedings of the National Academy of Sciences* **2021**, *118*.

(10) Garcia-Vidal, F. J.; Ciuti, C.; Ebbesen, T. W. Manipulating matter by strong

coupling to vacuum fields. *Science* **2021**, *373*, eabd0336.

(11) Genet, C.; Faist, J.; Ebbesen, T. W. Inducing new material properties with hybrid light–matter states. *Physics Today* **2021**, *74*, 42–48.

(12) Simpkins, B. S.; Dunkelberger, A. D.; Owrutsky, J. C. Mode-specific chemistry through vibrational strong coupling (or A wish come true). *The Journal of Physical Chemistry C* **2021**, *125*, 19081–19087.

(13) Baranov, D. G.; Munkhbat, B.; Zhukova, E.; Bisht, A.; Canales, A.; Rousseaux, B.; Johansson, G.; Antosiewicz, T. J.; Shegai, T. Ultrastrong coupling between nanoparticle plasmons and cavity photons at ambient conditions. *Nat. Commun.* **2020**, *11*, 2715.

(14) Yoshihara, F.; Fuse, T.; Ashhab, S.; Kakuyanagi, K.; Saito, S.; Semba, K. Superconducting qubit–oscillator circuit beyond the ultrastrong-coupling regime. *Nature Physics* **2017**, *13*, 44.

(15) Enkovaara, J.; Rostgaard, C.; Mortensen, J. J.; Chen, J.; Dułak, M.; Ferrighi, L.; Gavnholt, J.; Glinsvad, C.; Haikola, V.; Hansen, H., et al. Electronic structure calculations with GPAW: a real-space implementation of the projector augmented-wave method. *Journal of physics: Condensed matter* **2010**, *22*, 253202.

(16) Kuisma, M.; Sakko, A.; Rossi, T. P.; Larsen, A. H.; Enkovaara, J.; Lehtovaara, L.; Rantala, T. T. Localized surface plasmon resonance in silver nanoparticles: Atomistic first-principles time-dependent density-functional theory calculations. *Physical Review B* **2015**, *91*, 115431.
