# Supplementary material for: Polaritonic Chemistry from First Principles via Embedding Radiation Reaction
Source: J Phys Chem Lett. 2022 Jul 22;13(30):6905–11. doi: 10.1021/acs.jpclett.2c01169 (PMC9358701; doi:10.1021/acs.jpclett.2c01169)
Supplement: Supplementary file 2 — jz2c01169_si_002.pdf [file jz2c01169_si_002.pdf]

jz-2022-01169q.R1

Name: Peer Review Information for "Polaritonic Chemistry from First Principles via Embedding Radiation-Reaction"

First Round of Reviewer Comments

Reviewer: 1

Comments to the Author

This paper presents an application of an approach to simulating polaritonic systems that was recently co-proposed by the author. A radiation-reaction potential arising from a classical calculation of the electric field generated by the current from the moving quantum charges is applied back in the time-dependent Schroedinger equation of the charges; the relation between the field and the current-density is mediated by the classical dyadic Green's function. Although certainly interesting for both classical and quantized light-matter interactions, such as in polaritonic chemistry, I hesitate to recommend the current paper for publication in JPCLett, because of its closeness to the author's recently published PRL (Ref 47) on the subject: although the applications shown in the present work are different (although one of them, see below, seems very similar), there are not new methodological developments compared to Ref 47, nor particularly new insights/findings that would merit JPCLett, if I understood correctly. If I am mistaken, the author should make a better presentation of the novelty of the work; I also should note that the paper would benefit from a clearer re-writing as I found some of it a bit confusing.

The specific points are as follows:

(1) The abstract says that "Here we introduce an embedding approach…" but this paper is using the approach the author introduced in the recent Ref 47, so it is not really "introduced" here. Perhaps "apply" is more appropriate to use.

(2) End of the third paragraph: "This approach represents an extension of …" , please specify exactly what the extension is. Again, to me, it seems an application, rather than an extension, but perhaps I am mistaken.

(3) It might be useful to specify that the in-line Hamiltonian in paragraph 4, is an operator in the matter-space, i.e. that the light-fields are classical. (This is not apparent since the hat is placed only over the H but not over the operators on the right hand side).

(4) I am a bit confused about the general set-up for something like collective strong-coupling: does the approach work because of the locality of the interactions? That is, the field generated by the current is assumed to be non-zero only locally, and that fields from currents generated by other molecules in the ensemble are assumed not to contribute? Further, the locality appears even more extreme than this, since the two-point susceptibility is assumed to be nonzero only at coincidence (so a response at r only

when the perturbation is exactly at that same r). This seems to be a strong assumption. Is this necessary for the approach?

(5) TDDFT is used to compute the current from the time-dependent KS orbitals, but the KS current differs from the true current by a rotational component. This represents then an inherent approximation, and should be pointed out; References that discuss this and give explicit examples of the difference between these currents include, d'Agosta and Vignale Phys Rev B 71, 245103 (2005), Shaffhauser and Kuemmel Phys Rev B 93, 035115, and Dar et al. Phys. Rev. A 104, 03821 (2021).

Other approximations such as the exchange-correlation functional may have a larger effect but whether this is true is not known.

(6) To explain the computational procedure for the sodium atom chain, there is a description based on the flowchart in the TOC. It would be more helpful if specific equations were given. For example, it is said that first, the bare response of a single sodium dimer is obtained using the radiation-reaction potential, but this potential requires the current-density, but it is not said how that is obtained.

(7) The discussion around Fig 1 could be improved. First, I was not sure how to understand the figure in the context of the explanation regarding the scaling with N: it is said that the embedding approach describes the response of a single dimer, so has most spectral density in the dark state. But the figure is computing the photo-absorption of the whole chain, so how the red curve is exactly obtained from the response of a single dimer should be clarified. Further, it is not clear how the peak heights in the red. relate to, or are consistent with, the expected N-scaling of the response in the collective strong coupling regime; I see that the the direct calculation has amplitude increasing with N, while embedding has decrease. How does the ratio of the peak strength compare with what is expected from the collective being \sqrt(N) and having the dark states in the embedding?

(8) Also, I would say from this figure that the spectra are not particularly well-obtained by the approach, although the peak positions are good. I don't understand how the figure shows that the spectral weight is in excellent agreement as it is said in the caption.

Finally, Fig 3 of Ref 47 also studied this system; but the results look very different. Can the author clarify?

(9) In figure 3, how should we physically understand the N_ens = 0 results, that for small couplings, the effect does not increase with coupling strength (e.g. compare 0.0027 with 0.0054)?

I also did not understand the comment that with increasing ensemble size there is less effect of the cavity on the reaction since I thought that resonant collective coupling scaled as \sqrt{N}.

Also, overall in the figure, the numbers are very small -- the effect of the cavity seems very small, less than 0.1%.

(10) I am not sure what the author is wanting to convey in the sentence "An important realization is now that the mere necessity of the existence of collective

polaritonic states de nes a resonant condition". Is it that they mean the existence of collective polaritonic states defines a resonant condition? I thought that was already known. I am not sure what "mere necessity" is meaning in this sentence

Reviewer: 2

Comments to the Author

The author presents an embedding approach to extract electronic excitation energies with TD-DFT in the presence of a cavity that alters the response. The computational approach seems straightforward and believable and useful. In many ways, I want to recommend publication.

However, I am on the fence about this article for two reasons.

1. Although I think it is a good idea, I am not sure about exactly how new this approach is. On the one hand, the cavity angle may well be new. On the other hand, chemists have been embedding electronic structure in electromagnetic fields for a long time. For instance, PCM is incredibly old (though not frequency dependent). And what about ... THE JOURNAL OF CHEMICAL PHYSICS 138, 181105 (2013) and J. Chem. Theory Comput. 2020, 16, 9, 5695–5711, Now, the approaches above are time-dependent, and the author is proposing to do this in the frequency domain, but they are clearly relevant. To that end, I am just not sure what has been done, but it is also not clear to me that the author has thorougly researched what has been done exactly in this area and has cited the relevant (if any) papers.

2. It appears that the author wishes to model cavity effects in the dark (rather than the effect of irradiation on electronic structure). The author has then stipulated that the present approach may well explain the Ebbesen experiments, whereby a cavity can effect the rate of a reaction in a collective sense (i.e. the rate depends on the number of molecules in the cavity). But I cannot tell from the text whether the light-matter coupling (and volume) are reasonable. For instance, a set of g_0 values are listed on top of Fig. 3. Are these values realistic? And how exactly is g_0 entering the calculation?

Overall, it's an interesting article. With some improvement, it might be publishable in JPCL.

Author's Response to Peer Review Comments:

Chalmers University of Technology
Department of Microtechnology and Nanoscience - MC2

Chalmers MC2, SE-412 96 Gothenburg

Prof. Editor
The Journal of Physical Chemistry Letters

Christian Schäfer
Gibraltarvallsvägen 7
SE-412 96 Gothenburg
christian.schaefer.physics@gmail.com

Gothenburg, May 30, 2022

Dear Prof. Editor, dear referees,

Please find attached a revised manuscript (identifier jz-2022-01169q), a version with indicated changes as well as below a detailed response. The referees comments are repeated in italic, the response in plain text and implemented changes are highlighted in red.

I am grateful for the constructive and helpful comments that clearly improved the manuscript. Writing, but also evaluating, a publication with strong interdisciplinary character is challenging in my experience. I am confident that the below detailed response and the various improvements will answer all previous questions, clarify all misunderstandings and support the relevance and novelty of this work.

Referee 1:

This paper presents an application of an approach to simulating polaritonic systems that was recently co-proposed by the author. A radiation-reaction potential arising from a classical calculation of the electric field generated by the current from the moving quantum charges is applied back in the time-dependent Schroedinger equation of the charges; the relation between the field and the current-density is mediated by the classical dyadic Green's function. Although certainly interesting for both classical and quantized light-matter interactions, such as in polaritonic chemistry, I hesitate to recommend the current paper for publication in JPCLett, because of its closeness to the author's recently published PRL (Ref 47) on the subject: although the applications shown in the present work are different (although one of them, see below, seems very similar), there are not new methodological developments compared to Ref 47, nor particularly new insights/findings that would merit JPCLett, if I understood correctly. If I am mistaken, the author should make a better presentation of the novelty of the work; I also should note that the paper would benefit from a clearer re-writing as I found some of it a bit confusing.

I thank the referee for the detailed and constructive response. The revised manuscript accounts in many places for the referee's comments which greatly improved clarity.

However, I have to disagree with the referee's assessment. This manuscript does indeed introduce new methodology, i.e., the embedding component that introduces an ensemble of molecules into **G** via the Dyson equation and the from TDDFT obtained $\boldsymbol{\alpha}$. By virtue of this novel combination of techniques, the dynamic of a single molecule within a macroscopic state of collectively coupled molecules can be simulated. The systems shown in this manuscript are 'novel' since Ref. 47 did not show any results or considerations addressing collective strong (!) coupling. Not only is the novel approach introduced, its feasibility and accuracy demonstrated, but the manuscript collects further insight that suggests a nontrivial dependence of chemical reactivity on the number of collectively coupled molecules. To the best of my knowledge, the here presented methodology is the *only* available technique that might allow a *faithful* representation of the relevant experiments from *first principles* – a development that should merit publication in JPCLett. If this approach will recover or oppose experimental observations can only be speculated at this point but the here presented investigations add certainly an important and new perspective to the field.

The specific points are as follows: (1) The abstract says that "Here we introduce an embedding approach..." but this paper is using the approach the author introduced in the recent Ref 47, so it is not really "introduced" here. Perhaps "apply" is more appropriate to use.
(2) End of the third paragraph: "This approach represents an extension of ..." , please specify exactly what the extension is. Again, to me, it seems an application, rather than an extension, but perhaps I am mistaken.

Ref. 47 presents the basic idea how Maxwell's equation can be incorporated into time-dependent density-functional theory by usage of the radiation-reaction potential. It is vaguely stated that **G** could partially account for parts of the system by using an *ad hoc* macroscopic ε_r. However, this does not suffice to describe collective strong coupling from first principles, nor does it allow the scientific community to truly understand, use or validate the approach detailed in the present manuscript. Reference 47 sets its focus on memory-less emission in systems such as waveguides and free-space, i.e., weak coupling phenomena between an individual electronic system and a photonic environment. In this work, an extension is introduced that provides a clear step-by-step framework how collective strong coupling can be reformulated such that again only an individual molecular system has to be described explicitly and the remaining number of molecules are incorporated into **G**. Let me point out that the here used methodology demanded indeed notable work to be developed, implemented, thoroughly tested, applied and interpreted. Much of this effort is condensed in figure 1 and 3 or part of the supplemental information. Both, weak coupling/emission features and collective strong coupling are relevant in state-of-the-art scientific research. The former is highly relevant in many optical applications and thus addresses mostly physicists, while the latter gained recent interest as novel handle to control chemistry. Even though the initial concept, on which the

current manuscript bases the introduced extension, was published in a high impact journal, the extension is of even higher relevance for the polaritonic chemistry community. JPCLett has established itself as one of the major players in this field and presents therefore the ideal platform to present this manuscript. It sets out to present a novel technique that is able to fill a gap in the current landscape of research, sparks new ideas, opens perspectives and hopefully triggers new development in polaritonic chemistry and adjacent communities.

(3) It might be useful to specify that the in-line Hamiltonian in paragraph 4, is an operator in the matter-space, i.e. that the light-fields are classical. (This is not apparent since the hat is placed only over the H but not over the operators on the right hand side).

I thank the referee for noting this small aspect. The Hamiltonian has been adjusted accordingly.

(4) I am a bit confused about the general set-up for something like collective strong-coupling: does the approach work because of the locality of the interactions? That is, the field generated by the current is assumed to be non-zero only locally, and that fields from currents generated by other molecules in the ensemble are assumed not to contribute? Further, the locality appears even more extreme than this, since the two-point susceptibility is assumed to be nonzero only at coincidence (so a response at r only when the perturbation is exactly at that same r). This seems to be a strong assumption. Is this necessary for the approach?

Every molecule is contributing with its current and every molecule will 'feel' the field generated by all other molecules. However, under the common approximations that are widely believed to apply to collective strong coupling, the transverse far-field does not have any character of distance, i.e., the molecules can exchange energy via the photonic mode instantly and distance independent. Let me try to provide a slightly more detailed explanation in the hope that it will resolve the confusion, two aspects are especially important.

First, the basic interaction between (quantum) light and matter is local, as can be seen from the basic minimal coupling $\hat{H}_{pA} \propto \sum_i \hat{p}_i \hat{A}(\mathbf{r}_i)$. This implies that the susceptibility of independent particles is also local ($\chi(\mathbf{r},\mathbf{r}') \approx \chi(\mathbf{r})$). Only if light interacts with systems that are intrinsically strongly correlated over scales that are comparable with the optical wavelength (e.g. some plasmonic systems) can non-local susceptibility play a role. In our system and the experiments, the molecules are assumed to be dilute, i.e., they are only interacting via the common cavity mode. The polarizability $\alpha(\omega)$ describes how each of the individual molecules respond to a dipolar light field – this implies the long-wavelength approximation. In the vast majority of optical investigations, including collective strong coupling, the long-wavelength approximation $\hat{A}(\mathbf{r}_i) \approx \hat{A}$ is performed. This assumes that the full collection of matter that is coupling to the field is feeling the exact same field-strength. Clearly, this makes only sense if the wavelength of the field is much larger than the extension of the material. For molecules, this is usually very well satisfied as long as we stay in an non-relativistic energy-scale. Recall that

infrared light extends over μm, a considerable scale when compared to nm sized molecules. Quite fascinating is that even in solid-state physics this long-wavelength approximation is commonly and successful used, although a realistic solid should surely not satisfy this simple assumption. The momentum of photons is comparably small and the long-wavelength approximation sets it effectively to 0 – a good approximation in most cases. Surely it will break down when we reach relativistic effects such as Compton-scattering.

Second, the moment we perform the long-wavelength approximation for a system of molecules, we assume that they will all contribute in the same way to the photonic mode. However, this does not mean that the currents from different molecules will not contribute, it means exactly the opposite, each molecule will feel the field that is driven by all molecules. The cavity is thus introducing an effective all to all interaction. For classical light, the GS of the collective system is unaltered by the cavity, only the excited states will be affected. In the embedding approach, we perturb now a single molecule (e.g. the red one in the TOC graphic), the others are not directly feeling the perturbation. The red molecule will start to oscillate and emit into the cavity mode. Even though the other molecules have not been affected by the perturbation, they will respond now to the field that was generated by the red molecule. Their response will alter the field, recall that all molecules interact with the same cavity mode, and effect the red molecule. The simplification to purely local susceptibility and the long-wavelength approximation are not strictly necessary but they drastically simplify the routine. In this manuscript, I do not attempt to provide a general solution for light-matter interaction but a simple and computationally feasible framework for the description of collective strong coupling from first principles. The basic approximations, the target of this methodology and the limitations (e.g. no ultra-strong coupling) of this approach are discussed in length on page 2, 3 of the revised manuscript. Additional details and discussion are included in the SI.

(5) TDDFT is used to compute the current from the time-dependent KS orbitals, but the KS current differs from the true current by a rotational component. This represents then an inherent approximation, and should be pointed out; References that discuss this and give explicit examples of the difference between these currents include, d'Agosta and Vignale Phys Rev B 71, 245103 (2005), Shaffhauser and Kuemmel Phys Rev B 93, 035115, and Dar et al. Phys. Rev. A 104, 03821 (2021).

The referee is entirely correct that the KS current and the true physical current are not identical, I thank the referee for pointing out this confusing aspect of the current presentation. In this work and Ref. 47, we apply the commonly used long-wavelength approximation which allows us to express the integrated currents via the continuity equation as derivative of the dipole moment, in detail discussed in Ref. 47. The latter is by construction well reproduced by time-dependent DFT, as e.g. also stated in Dar et al. Phys. Rev. A 104, 03821 (2021). I acknowledge that the previous version failed to transfer this critical detail and apologize for the confusion.

The sentence 'The local radiation-reaction potential ...' does now specify that the derivative of the dipole moment is used and how it is related to the microscopic current.

(6) To explain the computational procedure for the sodium atom chain, there is a description based on the flowchart in the TOC. It would be more helpful if specific equations were given. For example, it is said that first, the bare response of a single sodium dimer is obtained using the radiation-reaction potential, but this potential requires the current-density, but it is not said how that is obtained.

The radiation-reaction potential of Ref. 47 and its extension, the embedding radiation-reaction potential, in this manuscript are TDDFT potentials that demand a self-consistent propagation of the time-dependent Kohn-Sham equations, as common in TDDFT. The current (as explained above) is reformulated into the derivative of the dipole moment, a quantity that is naturally obtained at every TDDFT cycle. Using the common predictor-corrector approach, the TD-Kohn-Sham equations are then solved hand-in-hand with the consistent local Kohn-Sham potential which depend on the electronic density. The form of the radiation-reaction potential is given on page 2. In order to improve clarity, the TOC graphic includes now an overview over the relevant equations and the computational procedure has been extended. The SI includes a series of simulation details. Furthermore, in case the referee is interested in very specific numerical details, I can recommend to review the publicly available qed-branch of gpaw `https://gitlab.com/christian.schaefer.physics/gpaw/-/tree/qed/`.

(7) The discussion around Fig 1 could be improved. First, I was not sure how to understand the figure in the context of the explanation regarding the scaling with N: it is said that the embedding approach describes the response of a single dimer, so has most spectral density in the dark state. But the figure is computing the photo-absorption of the whole chain, so how the red curve is exactly obtained from the response of a single dimer should be clarified. Further, it is not clear how the peak heights in the red. relate to, or are consistent with, the expected N-scaling of the response in the collective strong coupling regime; I see that the the direct calculation has amplitude increasing with N, while embedding has decrease. How does the ratio of the peak strength compare with what is expected from the collective being $\sqrt{N}$ *and having the dark states in the embedding?*

Understanding Fig. 1 is indeed essential to understand the embedding approach. The figure is supposed to clarify the difference between a direct simulation of the full chain (black lines) and the spectrum that the embedding approach provides for a single molecule out of the ensemble (red lines, see e.g. the sentence 'Note that the embedding approach describes the contribution of a single dimer to the collective state,...'). I thank the referee for pointing out potentially confusing aspects and I hope that the revised version is easier to digest. The SI includes further explanations and model calculations that support the understanding of this at first counter-intuitive behavior. Let me stress that the spectral response of both black and red lines are perfectly consistent and sensible as detailed below.

Fig 1 is presenting the photo-absorption spectrum of either the full chain (black lines) or only the single dimer (red lines), i.e., it combines two different 'responses'. The statement 'it is said that the embedding approach describes the response of a single dimer, so has most spectral density in the dark state.' is indeed correct. The embedding approach (red lines) describes the dynamic of a single molecule which is affected by presence of the environment comprising cavity + many other molecules. This means, that the embedding gives access to all excitations to which the single molecule contributes, i.e., even the dark states which are (by definition) invisible in the spectrum of the full chain (black lines). As pointed out by the referee, simulating the full chain leads to an increase of spectral intensity with N (as expected since more density is oscillating). However, the embedding approach will always only provide the dynamic of the single explicitly simulated molecules, i.e., its total spectral intensity is limited to the same amount of density, it just oscillates at different energies. Now, the more molecules contribute to the collective state (increasing N), the more dark states ($\sim N-1$) will emerge and the smaller the contribution of the single molecule in the collective state, as pointed out in the sentence 'The more molecules couple collectively, the smaller ($\propto 1/N$, e.g. (2 Na_2)/(4 Na_2) ≈ 2) the contribution of a single molecule.'. This is perfectly consistent with what we would expect from simple models such as the Tavis-Cummings model. The spectral response describes the density dynamic ($\mathcal{O}(\Psi^2)$) and the normalization of the collective states in those models $\frac{1}{\sqrt{N}}(|\phi_1\rangle|0\rangle + ...)$ is naturally introducing a $1/N$ decay of the contribution of the single molecule to the collective spectral response.

The revised manuscript includes now an extended discussion of Fig 1 to better account for the comments (7,8).

(8) Also, I would say from this figure that the spectra are not particularly well-obtained by the approach, although the peak positions are good. I don't understand how the figure shows that the spectral weight is in excellent agreement as it is said in the caption. Finally, Fig 3 of Ref 47 also studied this system; but the results look very different. Can the author clarify?

As described above, the spectra are not 1:1 transferrable to each other as the black lines describe the response of the full chain while the red lines describe the response of a single molecule in the collective state. Understanding the difference is key to understanding the embedding approach.

The comment regarding the excellent agreement of the spectral weight refers to two aspects. First, the slight asymmetry between upper and lower polariton is well reproduced by the embedding approach which suggests that detuning effects are well accounted for. Second, the trend of the spectral weight, i.e., black rising with N and red decreasing with $1/N$, is in excellent agreement with what is to be expected from simple models. I acknowledge however that this isolated statement might be confusing and apologize for the lack in precision. The sentence in the caption of figure 1 has been removed and instead a new sentence in the text has been added: 'Excitation energies and the asymmetry of the spectral weight are in excellent

agreement. As expected, the embedding approach recovers the with $1/N$ decreasing contribution of the single molecule to the collective state (see SI for an extended discussion).'

The systems are fundamentally different and thus the response looks different. In Ref. 47, the free emission of a small sodium chain is shown and weak coupling features are briefly discussed, i.e., the broadness of the excitation is relevant. Fig 1 presents however the spectrum for a chain which is coupled strongly and collectively to a single cavity mode and the relevant aspect is here the existence of polaritonic states with a given hybridization energy that separates them. Clearly, the physics in both systems is fundamentally different (weak vs strong coupling) which results in very different spectra.

(9) In figure 3, how should we physically understand the N_{ens} = 0 results, that for small couplings, the effect does not increase with coupling strength (e.g. compare 0.0027 with 0.0054)? I also did not understand the comment that with increasing ensemble size there is less effect of the cavity on the reaction since I thought that resonant collective coupling scaled as $\sqrt{N}$. Also, overall in the figure, the numbers are very small – the effect of the cavity seems very small, less than 0.1%.

Fig 3 includes now a subplot that presents results for up to $N_{ensemble} \approx 10^{11}$ and uses indeed relative cavity influence, clarifying that the effect is massive. The discussion has been revised and extended.

From the oscillating structure in this new subplot, it becomes apparent that the collective strong coupling is altering the proton-tunneling rate in this example in a nontrivial fashion and that the effect is likely to originate from energetic alignments that either favor or disfavor the overlap between the reactant and product states. In this case, there is no immediate reason to believe that the influence of the cavity on the reactivity should increase monotonically with the coupling strength. Since the cavity can feature both confining and transfer increasing aspects, it is *a priori* unclear how such a tunneling rate should be affected by the cavity. Notice that also the experiments have shown both catalysing and inhibiting effects of the cavity. However, it becomes clear from the domain ($N_{ensemble} = 10^4, N_{ensemble} = 10^6$) that larger fundamental coupling can indeed lead to a larger effect for *collective* strong coupling. Furthermore, this effect visible in the large $N_{ensemble}$ limit is inhibiting for all chosen fundamental coupling strengths and increases monotonically for larger coupling. This is consistent with experimental observations for vibrational strong coupling.

It is indeed a conundrum to the theoretical community why and how the influence on reactivity should scale linear with $\sqrt{N}$. As we discussed earlier, the contribution of a single molecule in the collective state decreases with $1/\sqrt{N}$, a specific molecule that attempts to undergo a reaction and is affected by the cavity should thus be less and less affected with increasing N. As the referee points out, current experimental work suggest however the opposite behavior. Fig 3 bottom does capture an effect like this for the weakest coupling (yellow) for small $N < 10$ but shows in the large N limit the opposite (theoretically more intuitive) trend. As stated in

multiple places, the presented model is clearly too simplistic to describe the complex chemical reactions in experiments. Nevertheless, it shows clearly that multiple different domains can exit and that the dependence on N is highly nontrivial, changing between increasing with N, to oscillating, to slowly decreasing with $1/N$. This conclusion is stated throughout the document and is, in my personal opinion, the only scientifically sound conclusion that one should transfer to the experimental setting. Any more specific claim, e.g., that the manuscript would confirm or oppose an experiment, would be highly speculative and conflicting with good scientific practice. Parts of the discussion have been reformulated to better account for this aspect. Please refer to the attached version in which changes are highlighted in red.

The 'Cavity influence on reactivity' is given as absolute, not relative, deviation. As shown in Fig 3 bottom, where the relative influence is presented (in %), the effect of the cavity is actually massive and easily on the same order of magnitude as the bare tunneling rate without cavity. Compare also the gray dashed with other lines in fig. 4 bottom. The word 'absolute' has been added to the definition of the cavity influence in order to prevent confusion and fig. 3 bottom and the according explanations clarify the magnitude of the catalyzing and inhibiting effect.

(10) I am not sure what the author is wanting to convey in the sentence "An important realization is now that the mere necessity of the existence of collective polaritonic states defines a resonant condition". Is it that they mean the existence of collective polaritonic states defines a resonant condition? I thought that was already known. I am not sure what "mere necessity" is meaning in this sentence

The sentence was supposed to convey the concept that one needs collective strong coupling in order to observe any effects. In order to obtain collective strong coupling, the cavity frequency has to be resonant with e.g. some vibrational mode of the reactant. This represents a first resonant condition, i.e., it is necessary to have collective strong coupling which implies a resonant condition. One could of course ask the next logical question 'To which of the available resonances should I tune my cavity in order to produce the strongest effect on the reactivity?'. As the referee states, this is indeed partially known from experimental research. However, it remains debated in theoretical work (e.g. Nat Commun 12, 1315 (2021)., J. Chem. Phys. 156, 154305 (2022). and all cited work). The sentence in question has been removed for brevity. It is not essential for this work and obstructed the flow of the manuscript.

I thank the referee again for the multitude of detailed and constructive comments.

Referee 2:

Comments: The author presents an embedding approach to extract electronic excitation energies with TD-DFT in the presence of a cavity that alters the response. The computational approach seems straightforward and believable and useful. In many ways, I want to recommend publication. However, I am on the fence about this article for two reasons.

I thank the referee for his constructive and positive response. I acknowledge that the presentation could be improved and hope that the performed changes suffice to clarify formerly opaque statements.

1. Although I think it is a good idea, I am not sure about exactly how new this approach is. On the one hand, the cavity angle may well be new. On the other hand, chemists have been embedding electronic structure in electromagnetic fields for a long time. For instance, PCM is incredibly old (though not frequency dependent). And what about ... THE JOURNAL OF CHEMICAL PHYSICS 138, 181105 (2013) and J. Chem. Theory Comput. 2020, 16, 9, 5695-5711, Now, the approaches above are time-dependent, and the author is proposing to do this in the frequency domain, but they are clearly relevant. To that end, I am just not sure what has been done, but it is also not clear to me that the author has thoroughly researched what has been done exactly in this area and has cited the relevant (if any) papers.

The fundamental embedding idea to embed parts of the systems into effective potentials or fields is of course not new, PCM being routinely used to account for solvation. The two additional references are interesting approaches to account for near-field effects in an elegant fashion. However, to my understanding, all references provided by the referee discuss near-field effects, i.e., aspects that emerge from the *short-range longitudinal* Coulombic components and the Poisson equation.

In contrast, this manuscript introduces an embedding approach that accounts for the *long-range transversal* ('light') interaction between far distant molecules (dilute ensemble). The Green's tensor accounting for the transverse fields is not the typical Coulombic 1/|r-r'| but allows almost distance independent interactions that are mediated by a limited set of Cavity modes. Those eigenmodes are the consequence of the boundary conditions, e.g., given by two mirrors building a Fabry-Perot cavity, that specify the solution of the Helmholtz equation. This set-up has been used to control chemical reactivity in experiment (see introduction) and a feasible theoretical approach is currently in high demand. To the best of my knowledge, there exists no embedding technique that would compare to the here presented work. If the referee is aware of any relevant publication I would be of course thankful and happy to cite and discuss it.

Nevertheless, I agree with the referee in the point that the mentioned publications should be cited at the part of the manuscript where longitudinal interaction is briefly mentioned, e.g., 'Coulomb mediated couplings could be included in the longitudinal component of the

embedding radiation-reaction ansatz.' This sentence has been adjusted to 'Coulomb mediated couplings could be included in the longitudinal component of the embedding radiation-reaction ansatz or simply via commonly available techniques such as PCM or frozen-density embedding[66–68].'. I apologize for overseeing this small aspect. The pre-print version of this work, available at arXiv:2204.01602, did indeed discuss longitudinal and solvation effects in more detail and cited work regarding PCM. It was necessary to substantially shorten the manuscript in order to adhere to the Letter-style of JPCLett which resulted in removing essentially all discussion of the embedding of longitudinal fields.

2. It appears that the author wishes to model cavity effects in the dark (rather than the effect of irradiation on electronic structure). The author has then stipulated that the present approach may well explain the Ebbesen experiments, whereby a cavity can effect the rate of a reaction in a collective sense (i.e. the rate depends on the number of molecules in the cavity). But I cannot tell from the text whether the light-matter coupling (and volume) are reasonable. For instance, a set of g_0 values are listed on top of Fig. 3. Are these values realistic? And how exactly is g_0 entering the calculation?

While this manuscript intends to describe collective strong coupling phenomena and provides important conclusions based on a model that is designed to follow experimentally relevant behavior, it would be far fetched to claim that the present model would "well explain the Ebbesen experiments". In contrast, it is stated that "Identifying the specific influence of collective strong coupling on a chemical reaction remains an open and theoretically challenging problem." and "Already our here illustrated simple proton-tunneling model exhibited a non-trivial behavior with the number of emitters. We can expect that complex multi-step chemical reactions will further complicate this trend." The here discussed model is too "simple" to claim any quantitative resemblance with the experimental conditions. However, the here obtained conclusions can be transferred in a 'qualitative' spirit towards the experiment. As discussed for instance in arXiv:2104.12429, the chemical reactions in experiment are too complex for simple models.

Most importantly, the here presented methodology is capable to provide a solution to this problem as it allows to describe arbitrary ensemble size hand in hand with an *ab initio* description of the experimental reaction. Also the conclusions drawn from the model are highly relevant for the polaritonic chemistry community as they oppose common claim such as that simply increasing g would amplify the effect or that the effect should vanish quickly for larger ensembles as a single molecule will only contribute with $1/\sqrt{N}$.

The quantity $g_0 \propto 1/\sqrt{V}$ enters via the quantization volume of the cavity into the calculation of $\mathbf{G}_\perp$ (details in the text and the SI). In atomic units, $g_0 = \sqrt{\frac{\omega_c 4\pi}{2V}}$ such that the quantization volumes used in the manuscript are of the order of $V^{\frac{1}{3}} \sim 10^3\, a_0 \approx 50\, nm$. Vibrational strong coupling uses infrared cavity that would suggest a few μm distance between the mirrors. The

here employed fundamental coupling is thus about 1 to 2 orders of magnitude larger than in experiment which is still substantially closer to experimental values than most other theoretical calculations. How many molecules couple collectively in the experiments is highly debated and hard to estimate, see e.g. arXiv:2108.12244. Simple model calculations would suggest something on the order of $N_{ensemble} \approx 10^9$, as said, this should be taken with a pinch of salt. Since we investigate in fig 3 (bottom) various couplings over many orders of magnitude in $N_{ensemble}$, we can expect that the here used values are indeed reasonable. The exact coupling and number of molecules is unknown and under heated debate. For this reason, I believe that a discussion of those questions in the present manuscript is of little service to the reader and might only divert from the central aspects of this work.

I thank the referee's again for their constructive feedback and hope that the response answered all questions and scattered all concerns.

Further changes to improve the presentation and adhere to the style-guidelines of JPCL include:

1. removed section heading 'Introduction'
2. added short description of Supporting Information
3. adjusted citation-style in manuscript and SI
4. updated reference 47
5. adjusted TOC graphic to better represent the idea and flow of the embedding approach
6. moved TOC graphic to title page (conflicts regarding size between linked PDF [`http://pubsapp.acs.org/paragonplus/submission/toc_abstract_graphics_guidelines.pdf`] and the author guidelines [`https://publish.acs.org/publish/author_guidelines?coden=jpclcd`], please specify if further adjustments are required)
7. labeled figures in the SI as S1, S2, ...
8. numbered all pages in the SI as S1, S2, ...

Yours sincerely,

Christian Schäfer

jz-2022-01169q.R2

Name: Peer Review Information for "Polaritonic Chemistry from First Principles via Embedding Radiation-Reaction"

Second Round of Reviewer Comments

Reviewer: 1

Comments to the Author

The author has made significant effort to address the concerns from the first round of reviewing. I appreciate that the first-principles approach presented could open the door to making feasible calculations of collective strong coupling in real systems, and shed light on some of the current controversies regarding mechanisms of processes in cavity-controlled chemistry. However before I would be able to recommend it for JPC Lett, there are points that I would need to have addressed as follows.

The author clarified in the response that the novelty lies in applying the method of Ref 47 to study collective strong coupling phenomena. Again, the wording in the abstract should better reflect that, rather than "introduce an embedding approach" since this approach was already introduced in Ref 47.

In his response, the author gives arguments regarding the assumption of the local susceptibility. First, he points to the relation H_{pA} = p.A(r) as being local, however this misses the point: the vector potential is evaluated at r but A has contributions generated from charges far away. That is, A depends non-locally on the charges. The discussion in the response is a bit contradictory since later it is stated that it is a simplification. A clearer discussion of what is the fundamental approximation is needed: is it the long wavelength approximation, or is the local approximation a separate approximation or does it follow? The argument given seems to conflate the two. In any case, taking the susceptibility as proportional to a delta function in space means that the response of the density at point r is unaffected by changes in the potential/density at any point other than r. This strikes me as a very significant approximation and an unusual one; even in approximations like LDA it is not the case that the entire susceptibility is approximated that way, only the exchange-correlation portion is.

Although the author has clarified Figure 1, a key demonstration of the effectiveness of the approach is missing from the figure. The point of the embedding approach would be to capture the black result (direct response calculation) with that obtained from embedding. Thus a plot that shows how the embedding calculation then yields something close to the exact curve is needed, i.e. incorporating the single dimer result in the method to approximate the black curve. This is not evident from the red curves shown which show the contribution of the single embedded molecule. It would be a strong demonstration of how good the embedding method is, if the benchmark black curve was reproduced by the embedding approach; can this information be extracted from the response of the embedded single dimer and how well does it perform?

Author's Response to Peer Review Comments:

Chalmers University of Technology
Department of Microtechnology and Nanoscience - MC2

Chalmers MC2, SE-412 96 Gothenburg

Prof. Editor
The Journal of Physical Chemistry Letters

Christian Schäfer
Gibraltarvallsvägen 7
SE-412 96 Gothenburg
christian.schaefer.physics@gmail.com

Gothenburg, July 4, 2022

Dear Prof. Editor, dear referees,

Please find attached the revised manuscript (identifier jz -2022-01169q). A detailed response as well as a version with indicated changes is found below. The referees comments are repeated in italic, the response in plain text and implemented changes are highlighted in red.

Referee 1:

The author has made significant effort to address the concerns from the first round of reviewing. I appreciate that the first-principles approach presented could open the door to making feasible calculations of collective strong coupling in real systems, and shed light on some of the current controversies regarding mechanisms of processes in cavity-controlled chemistry. However before I would be able to recommend it for JPC Lett, there are points that I would need to have addressed as follows.

I am thankful for the referee's effort in the form of many constructive and detailed comments and appreciate the scientific exchange that is unfortunately not common practice. Since the previous revision seemed to have resolved most concerns and confusions, I am confident that this new revision is sufficient to eliminate all remaining concerns and shape the publication into a state that aligns well with the interests of the Journal of Physical Chemistry Letters.

The author clarified in the response that the novelty lies in applying the method of Ref 47 to study collective strong coupling phenomena. Again, the wording in the abstract should better reflect that, rather than "introduce an embedding approach" since this approach was already introduced in Ref 47.

As stated before, Ref 47 focuses on the fundamental idea but refers to this manuscript for the **collective strong** coupling. From my perspective, the word "introduce" is perfectly adequate. The abstract has been adjusted to "Here, we demonstrate ..." in order to resolve this futile debate.

In his response, the author gives arguments regarding the assumption of the local susceptibility. First, he points to the relation H_{pA} = p.A(r) as being local, however this misses the point: the vector potential is evaluated at r but A has contributions generated from charges far away. That is, A depends non-locally on the charges. The discussion in the response is a bit contradictory since later it is stated that it is a simplification. A clearer discussion of what is the fundamental approximation is needed: is it the long wavelength approximation, or is the local approximation a separate approximation or does it follow? The argument given seems to conflate the two. In any case, taking the susceptibility as proportional to a delta function in space means that the response of the density at point r is unaffected by changes in the potential/density at any point other than r. This strikes me as a very significant approximation and an unusual one; even in approximations like LDA it is not the case that the entire susceptibility is approximated that way, only the exchange-correlation portion is.

The long-wavelength approximation for the ensemble is the fundamental approximation here, i.e., all molecules couple with the same strength to the field. Two points of the ensemble connected by a field A(r) = A($r_{reference}$) = A will only feel global operators (such as the position $\hat{V} \propto \hat{\mathbf{r}} \cdot \mathbf{E}_{\perp}$) and they will only couple to the field via their locally integrated currents $\mathbf{J}$. Consequently, a molecule at point 1 and a molecule at point 2 will interact via the transverse field only via their dipole polarizabilities $\alpha(\omega)$. The statement "A depends non-locally on the charges" is misleading. A does no longer have any sense of locality in this case, any current couples to the same global field A. The long-wavelength approximation for the ensemble eliminates all spatial resolution – from the perspective of the electromagnetic field all molecules inside the cavity are located at the exact same reference position $r_{reference}$. This is a common approximation and used throughout all theoretical approaches in polaritonic chemistry.

Furthermore, we assume subsequently that the ensemble is dilute, i.e., their intermolecular distance is so large that dipole-dipole interactions via the Coulomb interaction are negligible. This is motivated by the experimental conditions and widely used in this field. Extensions to consider Coulomb mediated interactions are briefly mentioned (e.g. combining the here presented approach with PCM-like approaches). If the molecules would sit much closer together, their interaction would introduce a deviation, as demonstrated in Fig. 1 (main text, comparing black to magenta line). Assuming infinite separation at the same time as assuming small size of the ensemble might seem contradictory but is consistent when considering the different length-scales we are working with. Infrared cavities feature μm wavelength's while Coulomb-mediated interactions decay over a length of nm. The embedding approach is in principle more general, we could for instance retain a position dependence for $\chi_E(\mathbf{r}, \omega)$ but this would deviate from the thus far investigated domain and open a new direction. In my humble opinion, the development of first-principles techniques for polaritonic chemistry should be done step by step. This suggests to start at a point for which we posses intuition in order to understand the nature of the developed embedding approach.

The dilute+dipolar ensemble assumption is conceptualized in the step $\chi_E(\mathbf{r}'', \omega) \approx \chi_E(\mathbf{r}_0, \omega)\theta(\mathbf{r} \in V_E)$ and transferred into a more "convenient" form $\approx \chi_E(\mathbf{r}_0, \omega)V_E\delta(\mathbf{r}-\mathbf{r}_0)$. The sentence clar-

ifies with "For a dilute ensemble satisfying the long wavelength approximation, ..." that this approximation is valid only within the long-wavelength/dipolar approximation. Subsequently, the equation characterizing $\mathbf{G}_\perp$ and with it the field $\mathbf{E}_\perp$ is purely global, the position is only appearing implicitly via the reference-position $\mathbf{r}_0 = \mathbf{r}_{reference}$.

It is important to distinguish macroscopic susceptibility (coupling via Maxwell's equation on the scale of the field) and the density-density response describing local changes in the density induced by local potentials (used commonly in TDDFT). As highlighted in the main text, both are related but fundamentally different. The density-density response $\chi_{\rho\rho}(\mathbf{r}, \mathbf{r}', \omega)$ of a single molecule has still its full complexity, the performed calculations use regular TDDFT with the PBE potential. From the perspective of the dipolar field, all those details vanish and only the macroscopic susceptibility of the ensemble remains.

Both approximations have been thoroughly described in the manuscript and are standard approximations in polaritonic chemistry. Any extensions are certainly interesting but beyond the scope of this work. The 'polaritonic chemistry' domain has been further emphasized in the revised version, the later simplifications are understood in this context.

Although the author has clarified Figure 1, a key demonstration of the effectiveness of the approach is missing from the figure. The point of the embedding approach would be to capture the black result (direct response calculation) with that obtained from embedding. Thus a plot that shows how the embedding calculation then yields something close to the exact curve is needed, i.e. incorporating the single dimer result in the method to approximate the black curve. This is not evident from the red curves shown which show the contribution of the single embedded molecule. It would be a strong demonstration of how good the embedding method is, if the benchmark black curve was reproduced by the embedding approach; can this information be extracted from the response of the embedded single dimer and how well does it perform?

I thank the referee for this important question.

This work focuses indeed on the single molecule affected by the collective coupling rather than the collective state itself. Critical features that should be captured are therefore the polaritonic resonances (as the single molecules should oscillate at the same frequency as the collective state), the ratio between bright and dark states and any asymmetry between polaritonic states. Ideally, we would isolate the dynamic of a single sodium dimer in the black curves. For the moment, comparing collective to embedded state is sufficient as it also illustrates their conceptual difference – an important pedagogical aspect of this work. Furthermore, isolating the local dipole moment from the black curves would demand some more in-depth coding in GPAW. The implementation will be further developed (e.g. to account for a more generic Green's tensor and nuclear motion) but provides already in its current form a conceptually new and exciting direction.

Nevertheless, it is entirely possible to obtain accurate approximations of the collective state response – *even if it is not the focus of this work and thus only of marginal relevance for its publication*.

The poles at which the embedded molecule oscillates coincides with the collective state resonances, the collective state (black line) can be reconstructed in two ways.

Option 1 is based on our knowledge about the system of identical emitters (obtained e.g. via the intuition obtained from the Tavis-Cummings model): The ratio between bright and dark states in the polarizability per molecule scales as $\frac{2}{N}$ for bright states vs $\frac{(N-1)^2}{N}$ for dark states. Since N identical molecules oscillate, a factor N has to be added. Multiplication of the bright states of our single (embedded) molecule by $2+(N-1)^2$ provides therefore a very good estimate of how strong the bright states of the collective state will be. This simple connection, emerging purely from the re-scaling of spectral density, provides already quite accurate results shown below.

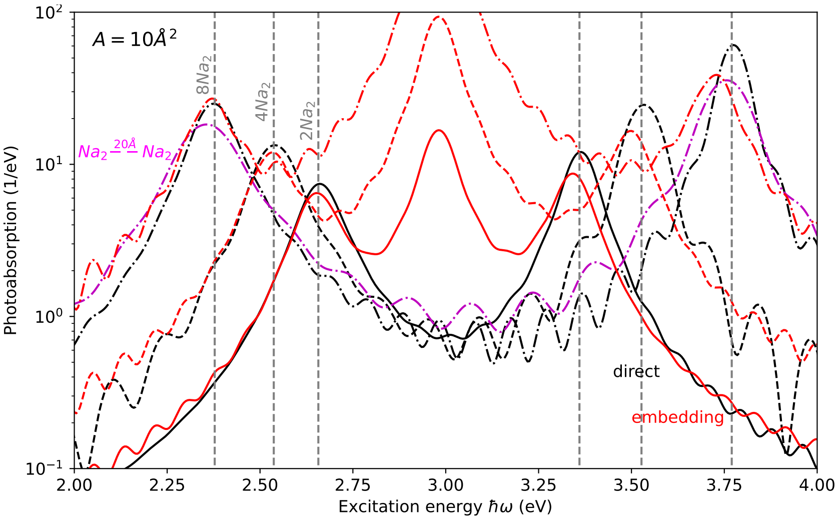

The dark states do no longer exist in the direct (black) calculation, i.e., we would in principle need to cut those states from the spectrum. Clearly, if the embedding environment becomes more complex, such an intuitive and model-inspired explanation becomes no longer reliable. Option 1 neglects the fact that for a more general system, the actually measured spectrum due to absorption/transmission will of course not only consist of the sum of simply re-scaled dipoles but must account for the correct electromagnetic emission. What we measure is not a dipole moment but the radiated/absorbed electric field.

Option 2 provides now the rigorous approach: We know that the radiated field is given by

$$\mathbf{E}_\perp(\omega) = i\mu_0\omega\mathbf{G}_\perp(\omega)\cdot(-i\omega)\mathbf{R}(\omega)$$
$$= \mu_0\omega^2\mathbf{G}_\perp(\omega)\cdot\alpha(\omega)\cdot\delta\mathbf{E}\ .$$

The multiplication with the full Green's function (including N times the single molecule α) weights correctly bright states and eliminates dark states. Clean spectra will demand a high frequency resolution and since the cavity is only weakly decaying in our example (we want strong coupling), very long propagation times would be necessary. This is circumvented in Fig. 1 (main manuscript) by artificial broadening. In order to take the above multiplications with sufficient accuracy and consistent norm, much higher resolution would be needed – especially for the direct approach (black lines). Nevertheless, by again artificially broadening the dipole response we can provide a proof-of-principle calculation shown below. The figure shows the polarizability, Green's tensor and radiated fields for direct (black) and embedding (red) approach. As elaborated in the SI, the frequency resolution for $\mathbf{G}_\perp$ is increased and well converged in our calculations but for the multiplication it is necessary to project back on the corse grid on which $\mathbf{R}(\omega)$ is given. The radiated fields are consistent, a more quantitative comparison would demand a higher resolution for the direct (black) calculation. Especially the high of upper and lower polariton are sensitive to the resolution and broadening as the multiplication with Green's tensor 'cuts' out only a part of $\alpha(\omega)$. Figure 1 in the main manuscript is here much more reliable as the direct use of the strongly broadened polarizability results the impact of low frequency resolution. Black lines use a propagation-time of 78.5 fs while the red (embedding) calculations allow much longer propagation such as 340 fs. Overall, the dark states are eliminated and upper and lower polariton are recovered (besides deviations due to the broadening).

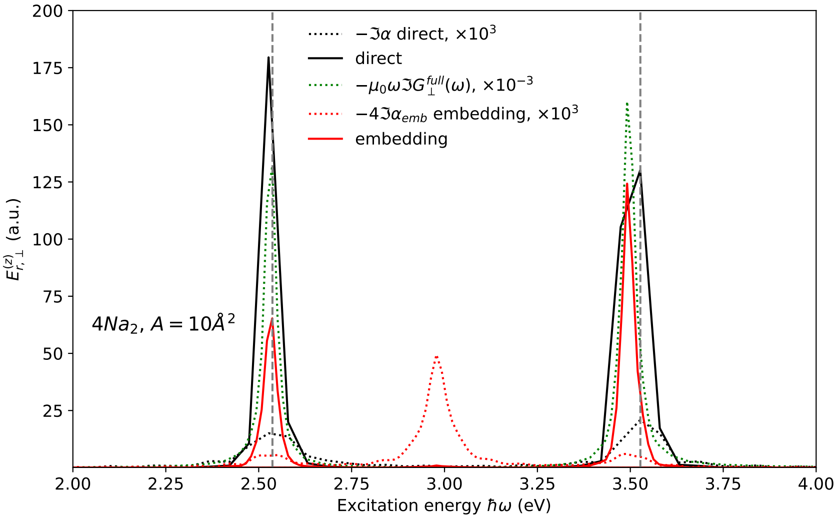

As pointed out before, the focus of this work is *not* to find a new way to describe collective polaritonic states. Instead, it lies in the goal to find an efficient approach to describe how a single molecule will evolve in such a collective state. The point is that chemical reaction will evolve locally molecule-per-molecule and the collective polaritonic states provide a new environment that is partially steering this dynamic. Nevertheless, the collective states can be recovered from the embedding approach – even if it is not the focus of this work.

Figure 1 (main manuscript) remains an important part of this work as it clarifies all conceptual differences between collective directly calculated spectra (black) and the embedding approach (red). I deem it pedagogically important to challenge the reader here a bit in order to clarify that the embedding approach is designed to deliver the dynamic of a single molecule that is embedded into the electromagnetic environment of many molecules coupled collectively via a cavity mode. The compact discussion is designed to fit to the format of a letter, additional details can be found in the SI.

I acknowledge, however, that the connection between red and black lines can be further elaborated. The SI discusses the above aspects now in detail and the main manuscript is adjusted to accommodate for this discussion in a brief fashion.

Irrespective of the above discussion. The demonstrated embedding approach reproduces accurately the critical features of collective strong coupling from the perspective of a single molecule. All observations around figure 1 are consistent with the existing knowledge and models. Embedding radiation-reaction is the only thus far available technique that provides realistic opportunities to faithfully model large ensembles used in polaritonic chemistry from first-principles.

I thank the referee again for the constructive feedback.

Further additions to solidify the conclusions include:

1. moved the sentence addressing the dipole-dipole induced blue-shift and PCM connections to a better position to avoid redundant text that obstructed the text-flow
2. in SI: added a detuning-series for only the frequency of the cavity
3. in SI: added a plot where the proton transfer is integrated for different times, illustrating the negligible sensitivity to changing T
4. in SI: added an example figure for the frequency resolution as well as some additional explanation

Yours sincerely,

Christian Schäfer
